# Inhibition of Methyltransferase DOT1L Sensitizes to Sorafenib Treatment AML Cells Irrespective of *MLL*-Rearrangements: A Novel Therapeutic Strategy for Pediatric AML

**DOI:** 10.3390/cancers12071972

**Published:** 2020-07-20

**Authors:** Annalisa Lonetti, Valentina Indio, Maria Antonella Laginestra, Giuseppe Tarantino, Francesca Chiarini, Annalisa Astolfi, Salvatore N. Bertuccio, Alberto M. Martelli, Franco Locatelli, Andrea Pession, Riccardo Masetti

**Affiliations:** 1 “Giorgio Prodi” Cancer Research Center, University of Bologna, S. Orsola-Malpighi Hospital, Via Massarenti 11, 40138 Bologna, Italy; valentina.indio2@unibo.it (V.I.); giuseppe.tarantino6@unibo.it (G.T.); or annalisa.astolfi@unife.it (A.A.); andrea.pession@unibo.it (A.P.); 2 Department of Experimental, Diagnostic, and Specialty Medicine, University of Bologna, S. Orsola-Malpighi Hospital, Via Massarenti 9, 40138 Bologna, Italy; antolaginestra3@gmail.com; 3 IRCCS Istituto Ortopedico Rizzoli, Laboratory of Experimental Oncology, via di Barbiano 1/10, 40136 Bologna, Italy; 4 Institute of Molecular Genetics, Luigi Luca Cavalli-Sforza—CNR National Research Council of Italy, 40136 Bologna, Italy; francesca.chiarini@gmail.com; 5 IRCCS Istituto Ortopedico Rizzoli, via di Barbiano 1/10, 40136 Bologna, Italy; 6 Department of Morphology, Surgery and Experimental Medicine, University of Ferrara, Via Luigi Borsari 46, 44121 Ferrara, Italy; 7 Pediatric Hematology-Oncology Unit, Department of Medical and Surgical Sciences DIMEC, University of Bologna, S. Orsola-Malpighi Hospital, Via Massarenti 11, 40138 Bologna, Italy; salvatore.bertuccio2@unibo.it (S.N.B.); riccardo.masetti5@unibo.it (R.M.); 8 Department of Biomedical and Neuromotor Sciences, University of Bologna, Via Irnerio 48, 40126 Bologna, Italy; alberto.martelli@unibo.it; 9 Department of Pediatric Hematology-Oncology and Cell and Gene Therapy, IRCCS Ospedale Pediatrico Bambino Gesù, Sapienza University of Rome, Piazza Sant’Onofrio 4, 00165 Rome, Italy; franco.locatelli@opbg.net

**Keywords:** pediatric acute myeloid leukemia, DOT1L, ChIP-seq, BRAF, targeted therapy, Pinometostat, Sorafenib

## Abstract

Pediatric acute myeloid leukemia (AML) is an aggressive malignancy with poor prognosis for which there are few effective targeted approaches, despite the numerous genetic alterations, including *MLL* gene rearrangements (*MLL*-r). The histone methyltransferase DOT1L is involved in supporting the proliferation of *MLL*-r cells, for which a target inhibitor, Pinometostat, has been evaluated in a clinical trial recruiting pediatric *MLL*-r leukemic patients. However, modest clinical effects have been observed. Recent studies have reported that additional leukemia subtypes lacking *MLL*-r are sensitive to DOT1L inhibition. Here, we report that targeting DOT1L with Pinometostat sensitizes pediatric AML cells to further treatment with the multi-kinase inhibitor Sorafenib, irrespectively of *MLL*-r. DOT1L pharmacologic inhibition induces AML cell differentiation and modulates the expression of genes with relevant roles in cancer development. Such modifications in the transcriptional program increase the apoptosis and growth suppression of both AML cell lines and primary pediatric AML cells with diverse genotypes. Through ChIP-seq analysis, we identified the genes regulated by DOT1L irrespective of *MLL*-r, including the Sorafenib target *BRAF*, providing mechanistic insights into the drug combination activity. Our results highlight a novel therapeutic strategy for pediatric AML patients.

## 1. Introduction

Acute myeloid leukemia (AML) is a genetically heterogeneous group of myeloid malignancies that account for approximately 20% of pediatric leukemias [1]. Currently, long-term cure is achieved in only 60% of pediatric AML cases and the possibility of relapse is still high, ranging between 25% and 35% [2], especially for poor prognosis AML subtypes including FMS-like tyrosine kinase 3 (*FLT3*) mutated and mixed lineage leukemia rearranged (*MLL*-r). Therefore, at present, improved therapies for pediatric AML remains an unmet need.

Recent studies have elucidated the role of the histone methyltransferase disrupter of telomeric silencing 1-like (DOT1L) in *MLL*-r leukemias. DOT1L is the only known methyltransferase catalyzing non-processive mono-, di-, and trimethylation of lysine 79 on histone H3 (H3K79), and the presence of H3K79me2 strongly correlates with active gene transcription [3]. DOT1L is a component of multiprotein complexes [4], and a number of proteins that it associates with are MLL fusion partners. As a result, in *MLL*-r leukemias, DOT1L is recruited on ectopic target sites, and its mislocation promotes the aberrant overexpression of developmentally important genes—for instance, *HOXA* cluster genes and *MEIS1* [5,6,7]. Accordingly, several studies have reported the role of DOT1L in leukemogenesis in the presence of different *MLL* rearrangements [6,8,9,10,11,12,13,14], and inhibiting DOT1L became an interesting strategy to impair leukemic transformation.

Pinometostat is a small molecule that blocks DOT1L activity by competing with the methyl-donor *S*-adenosyl-L-methionine (SAM). Due to the encouraging results obtained in preclinical studies [15], Pinometostat entered phase I clinical trials to treat both pediatric (NCT02141828) and adult (NCT01684150) refractory/relapsed patients affected by *MLL*-r leukemias, showing, however, modest clinical efficacy [16]. Moreover, additional leukemia subtypes, including *MLL*-PTD, *IDH1/2*-, *NPM1*-, and *DNMT3A*-mutated subtypes, were found to be sensitive to DOT1L inhibition [17,18,19,20,21], strengthening the importance of DOT1L methyltransferase activity for leukemic cells irrespectively of *MLL*-r. Several in vitro studies also demonstrated a combination benefit of DOT1L inhibitors in conjunction with chemotherapeutic agents or chromatin modifying drugs [22,23].

In light of the above, we hypothesized that DOT1L inhibition could sensitize pediatric AML cells to further treatment because of the impact on the regulation of genes involved in hematopoietic cell maintenance. In particular, we sought to investigate whether DOT1L inhibition with Pinometostat could sensitize AML cells, irrespectively of MLL fusions, to Sorafenib, a multi-kinase inhibitor with activity against FLT3, members of the RAF/MEK/ERK signaling pathway, VEGFR2, PDGFR, and KIT [24].

Our study demonstrates that combined treatment dramatically impacts AML cell proliferation and survival, despite the poor response to Pinometostat as a single agent and irrespective of *MLL*-r. We also tried to determine the mechanisms underlying Pinometostat sensitization of AML cells. Through ChIP-seq analysis, we identified the DOT1L targets shared between *MLL*-r and non-*MLL*-r AML cells that play relevant roles in cancer biology, including *RAF1* and *BRAF*, which encode for RAF/MEK/ERK signaling members inhibited by Sorafenib, and we validated the notion that strong BRAF down-modulation is a mechanism by which Pinometostat enhances Sorafenib efficacy. Collectively, our findings describe a promising approach to improve outcomes for pediatric AML patients.

## 2. Results

### 2.1. DOT1L Inhibition Impairs Proliferation and Induces Myeloid Differentiation Irrespective of MLL-r

**Figure 1:**
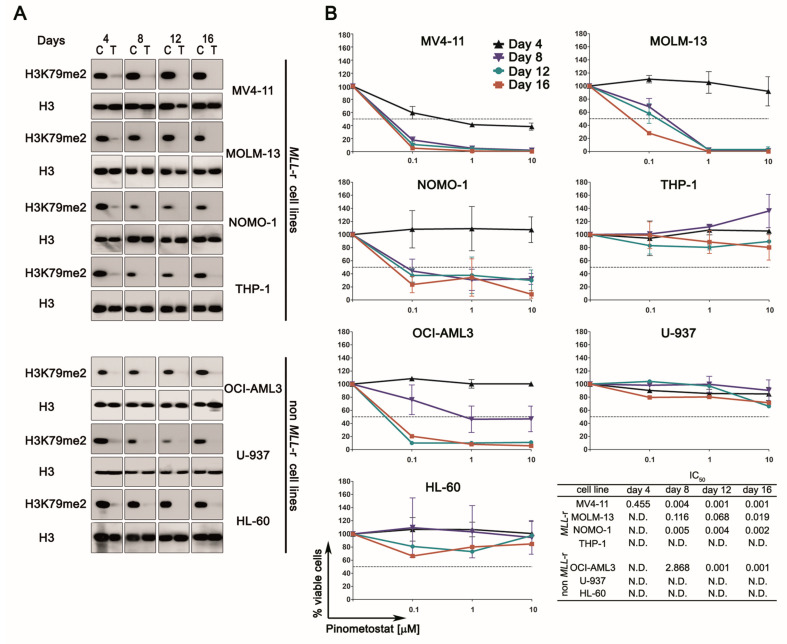
Pharmacological targeting of DOT1L in human acute myeloid leukemia (AML) cell lines with or without mixed lineage leukemia rearranged (*MLL*-r). (**A**) Western blotting analysis of histones isolated from *MLL*-r and non-*MLL*-r AML cell lines treated with 1 µM of Pinometostat or DMSO 0.01%. H3K79me2 levels were analyzed every 4 days for a period of 16 days. Molecular weights are indicated on the right. C: control cells; T: treated cells. (**B**) Growth curves of *MLL*-r and non-*MLL*-r AML cell lines treated with increasing concentrations of Pinometostat or DMSO 0.01% as vehicle control for up to 16 days and summary of IC_50_ concentrations. Results of three independent replicates are presented as means ± SD.

First, we tested the efficiency of 1 µM Pinometostat in inhibiting DOT1L methyltransferase activity, confirming that such a concentration, chosen so as not to exceed those used in vivo [16], caused the expected biological response, leading to the almost complete loss of H3K79me2 expression (Figure 1A). Since H3K79me2 loss impacts cell viability because of its involvement in regulating gene expression, we then examined the effect of Pinometostat on cell proliferation through flow cytometry to evaluate viable cell numbers (Figure 1B). As reported elsewhere, DOT1L inhibition impacted cell growth in the presence of *MLL*-r in a dose- and time-dependent manner. As expected, MV4-11, MOLM-13, and NOMO-1 cell lines, carrying MLL-AF4 or MLL-AF9 fusions, showed a dramatic decrease in cell number, with IC_50_ values below 1 µM following 8 days of treatment. In contrast, proliferation of U-937 or HL-60 cells, both lacking *MLL*-r, was unaffected by Pinometostat. Surprisingly, DOT1L inhibition had no effect on MLL-AF9-positive THP-1 cells, whereas it significantly decreased proliferation of the non-*MLL*-r OCI-AML3 cell line. These results demonstrate that the impact of DOT1L inhibition on cell proliferation is not strictly dependent on MLL fusions and suggest that additional factors could affect Pinometostat sensitivity. 

**Figure 2:**
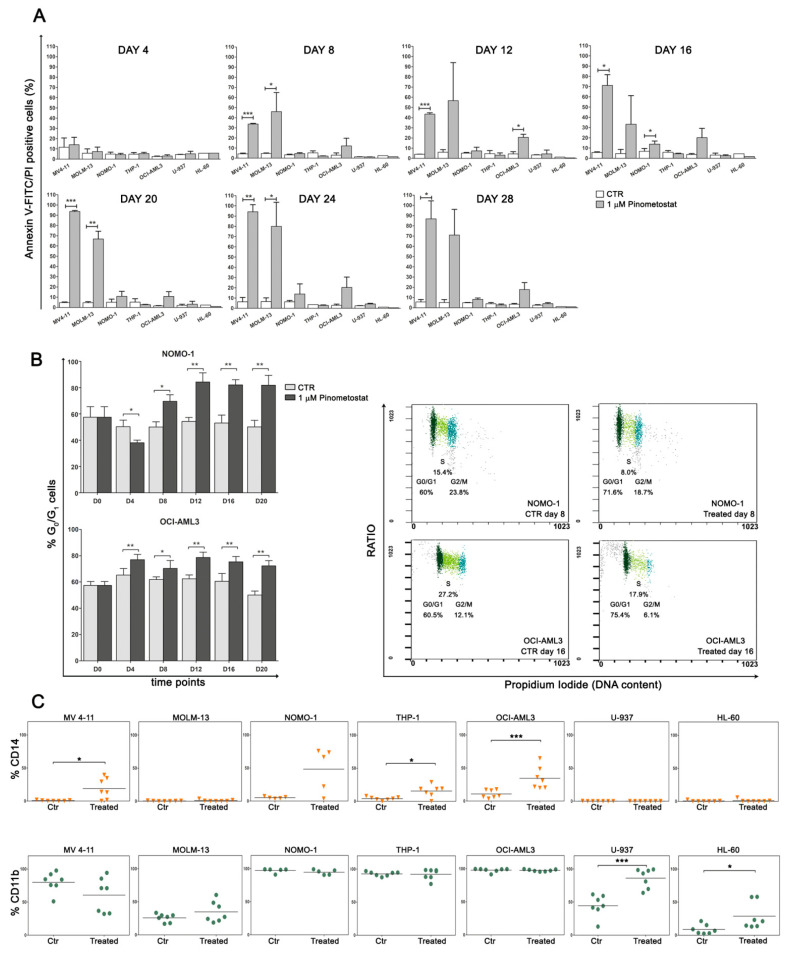
Impact of Pinometostat treatment on apoptosis, cell cycle, and differentiation. *MLL*-r and non-*MLL*-r human AML cell lines were treated with 1 µM of Pinometostat or DMSO 0.01% for up to 28 days and analyzed every 4 days. (**A**) Flow cytometry analysis of apoptosis measured by Annexin V-FITC/PI staining. Results of three independent replicates are presented as means ± SD. (**B**) Increase in G_0_/G_1_ phase cells in Pinometostat-sensitive NOMO-1 and OCI-AML3 cell lines, as measured by flow cytometry (left panels), and representative dot plots showing G_0_/G_1_ cell cycle phase increase at two time points (day 8 and 16, right panels). Cell cycle analyses were performed every 4 days for a period of 20 days (D0–D20). Results of two independent replicates are presented as means ± SD. (**C**) Modulation of CD14 (upper panels) and CD11b (lower panels) myeloid surface markers in AML cell lines. CD14 and CD11b were analyzed by flow cytometry every 4 days for a period of 28 days, and markers’ expression in Pinometostat-treated AML cell lines was compared to that of control cells. Ctr: control cells (DMSO 0.01%). Asterisks indicate levels of significance (* *p* ≤ 0.05, ** *p* ≤ 0.01, *** *p* ≤ 0.001).

Next, we assessed whether the proliferative disadvantage resulted from a cytotoxic or cytostatic response, observing that, in sensitive cell lines, reduced proliferation was accompanied by a significant induction of apoptosis only in MV4-11 and MOLM-13 cells (Figure 2A), while in treated NOMO-1 and OCI-AML3 cells, a progressive increase in G_0_/G_1_ phase cells was detected (Figure 2B). These findings strongly support the influence of the cellular environment, besides *MLL*-r, in determining cell fate following DOT1L inhibition.

We also monitored the expression of CD14 and CD11b myeloid-monocytic antigens, following prolonged exposure to Pinometostat, to assess whether DOT1L inhibition influenced cell differentiation. In all AML cell lines, these differentiation antigens were modulated over time, irrespectively of *MLL*-r and treatment responsiveness, with a statistically significant increase of at least one surface marker in most of the treated AML cell lines in comparison to untreated control cells (Figure 2C and Figure S1). We also verified that myeloid differentiation was promoted by Pinometostat and not caused by DMSO through a comparison of CD14 and CD11b expression in AML cell lines exposed or not exposed to the vehicle (Figure S2).

Overall, these results demonstrate that both cell death and cell cycle perturbation are consequences of DOT1L inhibition and appear to be dependent on cellular context rather than MLL fusions. In addition, because DOT1L inhibition influenced CD14 and CD11b expression in all AML cell lines, it can be assumed that DOT1L-mediated H3K79me2 regulates the expression of several genes involved in myeloid differentiation, and, consequently, the loss of this epigenetic marker induces terminal myeloid differentiation in AML cells, irrespectively of *MLL*-r. 

### 2.2. DOT1L Inhibition Impacts Proliferation Pathways Which Are Essential in Sustaining AML Cells

We next explored the biological effects of DOT1L inhibition on signaling pathways commonly involved in sustaining the proliferation and survival of AML cells.

*HOXA9* and *MEIS1* overexpression is a hallmark of *MLL*-r AML; however, their modulation did not appear to be associated with *MLL*-r or Pinometostat sensitivity. Indeed, a strong decrease in *HOXA9* transcript was detected in non-sensitive THP-1 and U-937 cell lines but not in responsive and *MLL*-r MV4-11 cells, whereas significant *MEIS1* down-modulation was a common mechanism resulting from DOT1L inhibition (Figure S3A). Since both *HOXA9* and *MEIS1* transcripts were not detected in the HL-60 cell line, we also ascertained the absence of vehicle-dependent mechanisms affecting gene expression (Figure S3B).

Since *FLT3* is regulated by HOXA9, and it is frequently highly expressed in AML cells (Figure S4), we evaluated the impact of DOT1L inhibition on the expression of *FLT3* and its key downstream components *STAT5a* and c-*MYC* (Figure S3A). H3K79me2 loss significantly reduced the amount of *FLT3* transcript from 4 days after drug treatment and without evident association with *MLL*-r. A similar expression pattern was also found for *STAT5a* and c-*MYC* genes. We further investigated whether Pinometostat treatment modulated multiple distal signaling pathways, including FLT3, PI3K/Akt, and MEK/ERK, which are frequently involved in sustaining the proliferation and survival of leukemic cells. We observed some impact on protein expression/activation only in a few cell lines (Figure S5A,B). Consistent with transcript quantification, DOT1L inhibition induced a reduction in total STAT5a protein, whereas the c-Myc immunoblot showed an increase in treated but unresponsive U-937 and HL-60 cells. By contrast, both PI3K/Akt and MEK/ERK pathways were functionally modulated by DOT1L inhibition, as pAkt, pErk, and pP38 decreased. However, these effects were modest and showed an uneven pattern that did not correlate with the presence of MLL fusions, neither with drug sensitivity nor drug exposure. Conversely, Pinometostat treatment resulted in a strong and continuous down-regulation of CDK6, an established DOT1L target [25], in all AML cells. These results demonstrate that, although Pinometostat treatment impacts multiple pathways, it is likely that DOT1L has an indirect role in regulating FLT3, PI3K/Akt, or MEK/ERK signaling.

### 2.3. Primary MLL-r AML Cells Are Barely Affected by DOT1L Inhibition

**Figure 3:**
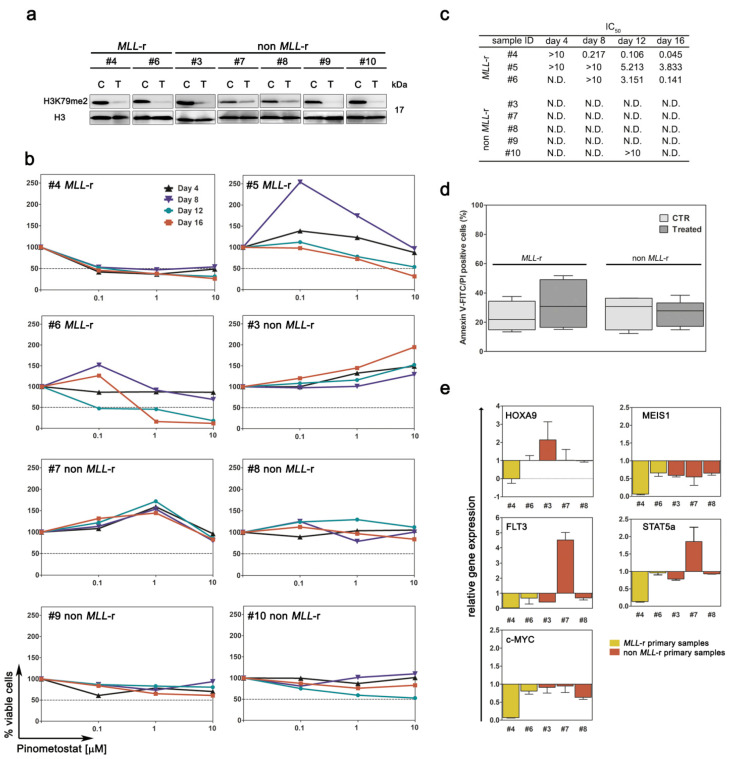
Pharmacological targeting of DOT1L in primary AML cells derived from pediatric patients with or without *MLL*-r. Primary AML cells were treated with Pinometostat for up to 16 days. (**A**) Western blot analysis of H3K79me2 levels following 8 days of treatment with 1 µM of Pinometostat or DMSO 0.01%. Molecular weights are indicated on right. C: control cells; T: treated cells. (**B**) Growth curves of primary AML cells treated with increasing concentrations of Pinometostat for up to 16 days. (**C**) Summary of IC_50_ concentrations. (**D**) Average percentage of apoptotic cells measured by flow cytometry in *MLL*-r and non-*MLL*-r primary AML cells treated for 16 days with 1 µM of Pinometostat or DMSO 0.01%. Ctr: control cells. Data are presented as means ± SD. (**E**) Modulation of gene expression induced by 8 days of treatment with 1 µM of Pinometostat and quantified by RT-PCR. Relative mRNA expression in treated vs. control cells was calculated using the 2^−ΔΔCt^ methods. Results of three independent replicates are presented as means ± SD.

We next aimed to determine Pinometostat activity in a clinically relevant context by analyzing ex vivo primary AML cells isolated from pediatric patients with or without *MLL*-r. To assess DOT1L inhibition, global levels of H3K79me2 were evaluated following exposure to Pinometostat (Figure 3A). Next, the sensitivity of primary AML cells to Pinometostat was investigated. As expected, none of the primary samples with wild type *MLL* exhibited a diminished proliferation. However, *MLL*-r primary samples were also poorly affected by the treatment, since cell growth impairment was limited to higher drug concentrations and longer exposure times (Figure 3B,C), and Pinometostat was unable to induce significant apoptosis even after 16 days of treatment (Figure 3D). These ex vivo results are in agreement with the modest efficacy of Pinometostat as a single agent, as observed in clinical studies on *MLL*-r leukemic patients [16]. In primary AML samples in which treatment with Pinometostat resulted in cell growth yielding sufficient RNA or protein, we also analyzed the impact of DOT1L inhibition on FLT3, PI3K/Akt, and MEK/ERK pathways by means of RT-PCR and immunoblotting. Similar to AML cell lines, treatment with Pinometostat affected gene expression irrespective of *MLL*-r. Indeed, *HOXA9* gene down-modulation was detected in one of the two analyzed *MLL*-r primary samples, whereas *MEIS1* mRNA levels decreased in all primary samples (Figure 3E). Conversely, a very poor impact on FLT3, PI3K/Akt, and MEK/ERK pathways was observed (Figure S6). Overall, these results demonstrate the limited efficacy of Pinometostat as a single agent in primary AML pediatric samples in spite of the presence of *MLL*-r.

### 2.4. DOT1L Inhibition Induces Gene Disregulation Not Limited to MLL-r Cells

In an attempt to better define the signaling pathways altered by DOT1L inhibition, we characterized global gene expression changes in both *MLL*-r and non-*MLL*-r cells treated with Pinometostat. 

**Figure 4:**
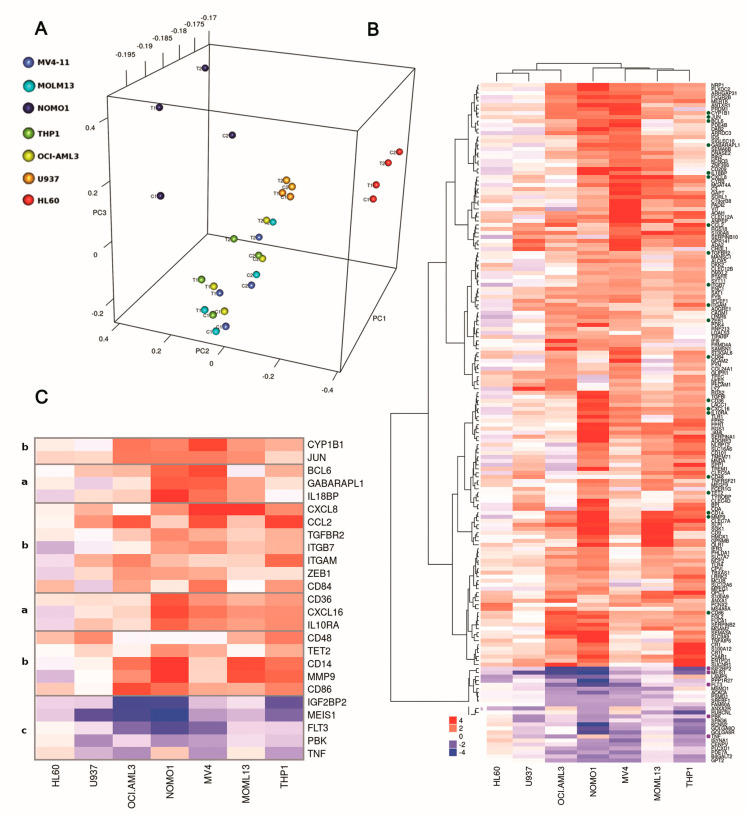
Global gene expression profiling in *MLL*-r and non-*MLL*-r human AML cell lines treated with Pinometostat. All AML cell lines were treated with 1 µM Pinometostat or DMSO 0.01% for 8 days, because in previous analyses, this drug exposure time significantly impacted proliferation, differentiation, and transcription processes. (**A**) Principal component analysis (PCA) plot of gene expression data. Two replicates for each cell line and condition are shown. C1: control cells, replicate 1; T1: treated cells, replicate 1; C2: control cells, replicate 2; T2: treated cells, replicate 2. (**B**) Heatmap and hierarchical clustering of fold changes in gene expression in response to Pinometostat for the 171 genes concordantly up- or down-regulated in at least three out of seven AML cell lines. The dendrogram clusters genes based on *MLL*-r. Genes involved in sustaining cancer cells or in hematopoietic cell differentiation are indicated with dots and are detailed in (**C**) (group a: up-regulated genes only in *MLL*-r cells; group b: up-regulated genes shared between *MLL*-r and non-*MLL*-r cells; group c: down-regulated genes shared between *MLL*-r and non-*MLL*-r cells). The color scale reflects the fold change in gene expression in response to Pinometostat, ranging from down-regulated (blue) to up-regulated (red).

Principal component analysis (PCA) of gene expression profiles revealed a main cluster comprising most of the AML cell lines and including both *MLL*-r and non-*MLL*-r cells (Figure 4A), thus indicating that treatment with Pinometostat did not induce high variation. Only HL-60 and NOMO-1 cells clustered separately. Then, we performed a paired sample analysis to identify differentially expressed genes upon DOT1L inhibition within each cell line (Table S3). A *p* value cutoff of 0.05 and a fold change of 1 were used to select a preliminary list of genes, and then we selected concordantly up- or down-regulated genes in at least three cell lines, thus obtaining a total of 171 genes, including 24 down- and 98 up-regulated genes in both *MLL*-r and non-*MLL*-r cells and 49 up-regulated genes in *MLL*-r cells (Figure 4B). To search for functionally important genes modulated by DOT1L inhibition, we performed a functional enrichment analysis for signaling pathways. The most enriched pathways included transcriptional misregulation in cancer and hematopoietic cell lineage, comprising genes which are essential in promoting growth and hematopoietic cell differentiation (Figure 4C). In particular, *MEIS1*, *FLT3*, *TNF*, *PBK*, and *IGF2BP*, all associated with cancer, were down-regulated. By contrast, the hematopoietic differentiation antigens CD14, CD36, CD48, CD84, and CD86 were up-regulated. Collectively, these findings confirm that DOT1L inhibition impacts the transcription of genes involved in leukemic cell survival and differentiation in both *MLL*-r and non-*MLL*-r AML cells.

### 2.5. Pharmacological Inhibition of DOT1L Sensitizes Both MLL-r and Non-MLL-r AML to Sorafenib Treatment

Due to the strong impact of DOT1L inhibition on proliferative signaling and the induction of myeloid differentiation irrespective of *MLL*-r, we hypothesized that treating both *MLL*-r and non-*MLL*-r AML cells with Pinometostat would induce sensitization to further treatment with the multi-kinase inhibitor Sorafenib.

As a single agent, Sorafenib reached a median IC_50_ of 6 µM after 48 h treatment (Figure S7A). To test our hypothesis, we performed drug combination studies according to a pre-treatment model in which cells were incubated for several days in the presence of Pinometostat prior to the addition of the second agent (Pinometostat/Sorafenib ratio 1:1). A range of clinically achievable drug concentrations for both Pinometostat and Sorafenib (0.1–10 µM) was employed [16,26]. In the majority of AML cell lines, combined treatment was more effective than each single agent (Figure S7B), with a median reduction in viable cells, among all AML cell lines, of at least 30%, already at low drug concentrations (Figure S7C). To determine the impact on proliferative pathways, immunoblot analysis was performed (Figures S7D and S8). Sorafenib exposure slightly impacted the activation or total protein expression of downstream FLT3 targets including STAT5a, STAT3, and cMyc in a few cell lines. Intriguingly, in MV4-11, NOMO-1, and U-937 cells, the combination reduced p-mTOR and p-S6RP, two key components of PI3K pathway, which in turn might be activated by FLT3, among others. 

**Figure 5:**
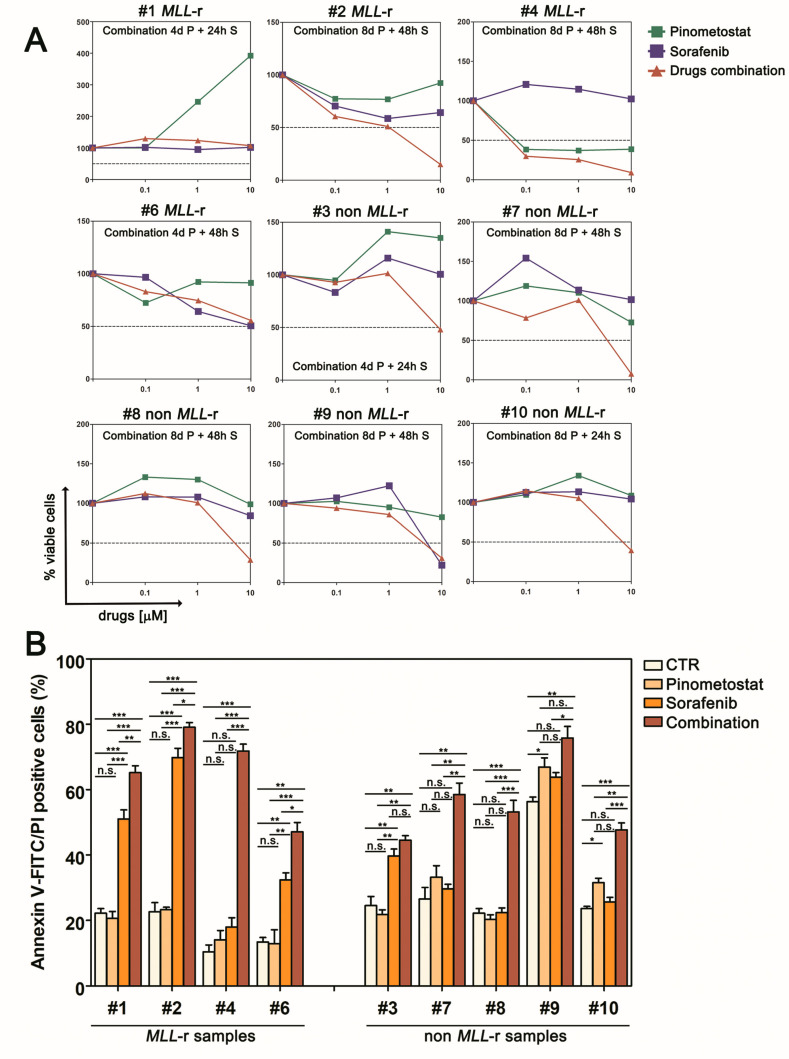
Pinometostat sensitizes primary cells from pediatric AML patients to Sorafenib treatment. (**A**) Growth curves of primary AML cells pre-treated with Pinometostat before Sorafenib addition (Pinometostat/Sorafenib ratio 1:1). The pre-treatment model consists of 4 or 8 days of treatment with Pinometostat followed by 24 or 48 h treatment with Sorafenib. Combination times are indicated in each panel. (**B**) Flow cytometry analysis of apoptosis measured by Annexin V-FITC/PI staining in primary AML cells treated with 10 µM of Pinometostat (8 days), Sorafenib (48 h), or combined drugs (8 days + 48 h). Ctr: control cells (DMSO 0.01%). Results of three independent replicates are presented as means ± SD.

We then assessed the efficacy of the Pinometostat/Sorafenib combination in pediatric AML primary samples. Enhancement of Sorafenib anti-proliferative activity was observed in six out nine primary samples following pre-treatment with Pinometostat (Figure 5A), although Sorafenib and Pinometostat as single agents led to a poor apoptotic response in those primary samples carrying *FLT3* mutations (samples #3 and #4) or MLL fusions (samples #1, #2, #4, and #6). Although the favorable interaction between Pinometostat and Sorafenib was not seen in all the primary samples - which is not surprising because of the high heterogeneity of AML- it should be noted that combined treatment was particularly effective in inhibiting the proliferation of non-*MLL*-r primary samples. In addition, analysis of Annexin-V positive cells mirrored this trend, demonstrating the co-treatment’s ability to increase apoptosis in comparison to both single agent treatments (Figure 5B). To validate the impact of DOT1L inhibition on enhancing sensitivity to Sorafenib irrespective of *MLL*-r, we analyzed nine additional *MLL* wild type samples, including four AML cell lines (Figure S9) and five primary pediatric AML samples (Figure S10). Although in some samples, including the KASUMI-1 cell line and sample #13, long-term exposure to Pinometostat as a single agent affected cell viability (Figures S9A,B and S10A), while combined treatment with Sorafenib decreased the number of viable cells (Figures S9A and S10B) and induced apoptosis (Figure S10C) relative to the single drugs. Collectively, these data demonstrate that sequential treatment with Pinometostat and Sorafenib enhances cytotoxicity over single drugs, and because this effect is not restricted to AML cells carrying Pinometostat or Sorafenib targeted genomic lesions, this drug combination provides the rationale for a novel treatment for pediatric AML.

### 2.6. DOT1L Target Genes Are Mainly Involved in AML Maintenance Irrespective to MLL Fusions

To better understand the role of DOT1L in AML cells, through ChIP-seq analysis, we mapped the genome-wide distribution of H3K79me2 in order to characterize genes that would be directly regulated by its methyltransferase activity.

**Figure 6:**
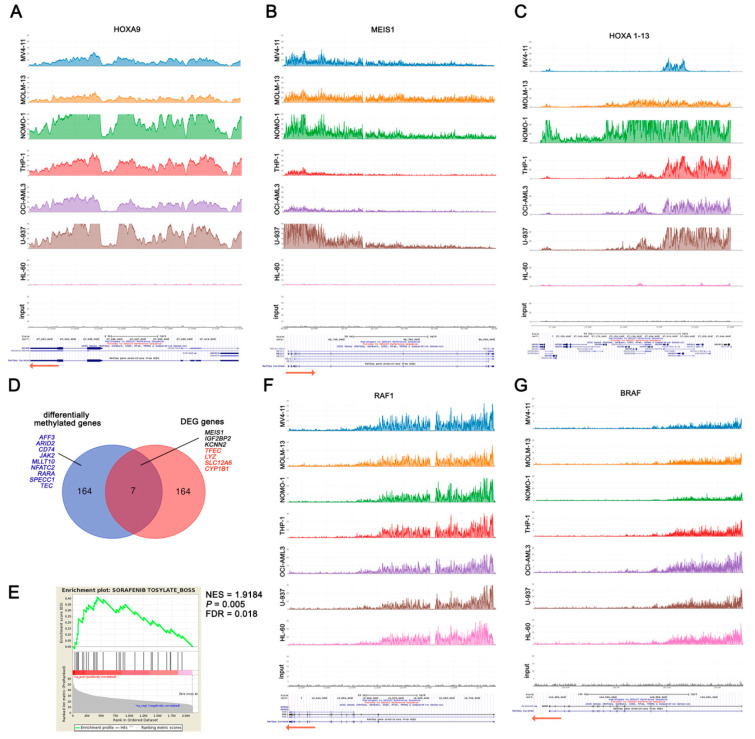
H3K79 methylation in *MLL*-r and non-*MLL*-r human AML cell lines. (**A**–**C**) are H3K79me2 profiles of MLL target genes. (**D**) Venn diagram showing the overlap between the 172 genes differentially methylated in *MLL*-r compared to non-*MLL*-r human AML cell lines and the 171 genes differentially expressed upon Pinometostat treatment and shared between at least three AML cell lines. Overlapping genes are reported (black: down-regulated genes; red: up-regulated genes). Additional genes with important roles in regulating signaling cascades, transcription, or cell development, and differentially H3K79me2 enriched in *MLL*-r compared to non-*MLL*-r, are indicated in blue. DEG: differentially expressed genes. (**E**) Gene set enrichment analysis (GSEA) of commonly H3K79me2 enriched genes in both *MLL*-r and non-*MLL*-r AML cells showing the enrichment in Sorafenib’s drug signature. The normalized enrichment score (NES) and the statistically significant False Discovery Rate (FDR) and *p* values are indicated. (**F**) and (**G**) are H3K79me2 profiles of *RAF1* and *BRAF*, which encode for Sorafenib targets.

First, we evaluated H3K79me2 enrichment of the MLL target genes *HOXA9* and *MEIS1*, and H3K79me2 was detected in all AML cell lines, excluding HL-60 cells (Figure 6A,B). To validate the ChIP-seq results, we performed qPCR analysis, which confirmed elevated H3K79me2 (>10% enrichment) upstream *HOXA9* and *MEIS1* in all AML cell lines except HL-60 cells (Figure S11). We next extended the analysis to the entire HOXA cluster, defining the core methylated region across all the cell lines, which included *HOXA9* and *HOXA10* genes, whereas the extension of H3K79me2 distribution in the entire cluster differed among AML cells in spite of their genomic features (Figure 6C). Notably, H3K79me2 enrichment of *HOXA9* and *MEIS1* or the number of HOXA loci with the H3K79me2 epigenetic mark was not associated with either *MLL*-r or the impact of DOT1L inhibition on proliferation and survival.

Relative to MLL fusions, we observed similar average levels of global H3K79me2 across the genome (Figure S12); therefore, in an attempt to identify genes that would be regulated as a result of mislocated DOT1L enzymatic activity, we conducted a strict comparative analysis between *MLL*-r and non-*MLL*-r cells, employing multiple bioinformatics tools to perform a differential binding analysis of ChIP-seq data. We identified 172 genes with a differential H3K79me2 peak enrichment, including 136 genes almost exclusively methylated or with at least a two-fold H3K79me2 peak enrichment in *MLL*-r cells, whereas only 18 genes had a two-fold H3K79me2 peak enrichment in non-*MLL*-r cells (Table S4). To better understand the relationship between H3K79me2 enrichment and gene expression, we compared the 172 genes with differential peak enrichment to the 171 differentially expressed genes upon DOT1L inhibition (selected as above described), detecting only seven shared genes (Figure 6D). In addition, we identified further genes involved in regulating signaling cascades, transcription, or cell development, with higher H3K79me2 in *MLL*-r compared to non-*MLL*-r, even if not down-regulated following DOT1L inhibition (Figure 6D). Overall, these data support a complex relationship between H3K79me2 amount and active gene transcription, suggesting that several factors besides MLL fusions cooperate with DOT1L to regulate gene expression.

To gain an insight into the relationship between DOT1L inhibition and AML cell sensitization to Sorafenib, we investigated which genes would be regulated by DOT1L irrespective of its MLL-fusion-dependent mislocation. We selected a common DOT1L target set of 2121 genes with elevated H3K79me2 levels shared among all AML cell lines (Table S5). We identified a multitude of genes that are relevant in sustaining the proliferation and survival of leukemic cells, including *DOT1L* itself (Table 1 and Figure S13). Notably, 190 out of 2121 DOT1L target genes were included in the Cancer Gene Census (CGC) (Table S5). Gene set enrichment analysis (GSEA) showed that this common DOT1L gene target set is significantly enriched in Sorafenib’s drug signature (Figure 6E). Therefore, we considered whether the favorable interaction of Pinometostat and Sorafenib could rely on DOT1L-dependent gene regulation of Sorafenib targets. *FLT3* was enriched in H3K79me2 only in several cell lines, and almost no H3K79me2 was detected in *PDGFRB*, *KIT*, *FGFR1,* and *VEGFR2* genes (Figure S13). By contrast, high peak enrichment was detected in both *RAF1* and *BRAF* genes in all AML cell lines (Figure 6F,G). Taken together, these data demonstrate that, irrespectively of MLL fusions, DOT1L is directly involved in regulating a multitude of genes implicated in cell proliferation and survival and suggest that Pinometostat treatment could enhance Sorafenib’s efficacy in both *MLL*-r and non-*MLL*-r leukemic cells by affecting the DOT1L-dependent regulation of Sorafenib target genes *RAF1* and *BRAF*.

### 2.7. DOT1L Inhibition Enhances Sorafenib Efficacy through Down-Modulation of BRAF 

We tried to further determine the biological mechanism by which Pinometostat sensitizes AML cells to Sorafenib. Since a consequence of DOT1L inhibition commonly observed in both *MLL*-r and non-*MLL*-r AML cell lines was myelomonocytic differentiation, we first analyzed whether the differentiation stage affected the response to Sorafenib treatment. To this end, all AML cell lines were exposed to PMA for 48 h, and PMA-induced adherent cells were tested for CD14 and CD11b expression to confirm myeloid–monocytic differentiation (Figure S14A). Subsequently, both undifferentiated and differentiated cell lines were exposed for 48 h to Sorafenib and were assayed by MTT. However, no significant differences were observed, and higher IC_50_ values than expected were detected with respect to the differentiation stage, suggesting that Pinometostat-dependent promotion of differentiation did not enhance sensitivity to Sorafenib (Figure S14B,C).

**Figure 7:**
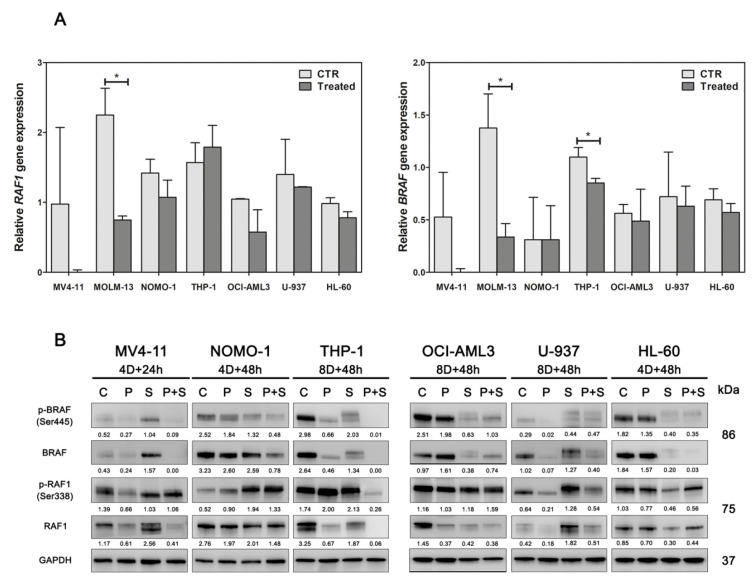
Impact of Pinometostat and Sorafenib on RAF1 and BRAF expression. (**A**) Assessment of *BRAF* and *RAF1* gene expression in AML cell lines treated with the DOT1L inhibitor Pinometostat for 16 days. CTR: control cells (DMSO 0.01%); Treated: cells treated with 1 µM Pinometostat. Results of three independent replicates are presented. Data are expressed as 2^ΔΔCt^ ± SD, and the universal human reference RNA was used as calibrator. (**B**) Western blot analysis of BRAF and RAF1 total and phosphorylated proteins in AML cell lines treated with 1 µM of Pinometostat and Sorafenib as single agents or in combination. Combination times are indicated for each cell line (4/8D: 4/8 days). Antibody to GAPDH served as a loading control. Molecular weights are indicated on right. C: control cells (DMSO 0.01%); P: Pinometostat treatment; S: Sorafenib treatment; P+S: combined treatment (Pinometostat and Sorafenib). Densitometric data were normalized on GAPDH.

As both *RAF1* and *BRAF* genes were identified as DOT1L targets, and they are also both targets of Sorafenib, another mechanism potentially responsible for the effectiveness of the Pinometostat/Sorafenib drug combination could be their stronger down-regulation. Therefore, to validate this hypothesis, we first investigated the impact of DOT1L inhibition on *RAF1* and *BRAF* expression, observing a trend toward a decrease in the transcript amount of these genes (Figure 7A). Then, we analyzed RAF1 and BRAF protein expression in AML cell lines treated with Pinometostat and Sorafenib alone or in combination (Figure 7B). We found a reduction in both RAF1 and BRAF proteins with Pinometostat as a single agent, confirming the role of DOT1L in RAF1 and BRAF regulation. Surprisingly, treatment with Sorafenib as a single agent affected the expression of total proteins, and in several cell lines, including MV4-11 and U-937, it increased protein levels in treated cells. In spite of this, the drug combination drastically reduced the expression of both total and phosphorylated RAF1 and BRAF, thus supporting our hypothesis. Finally, through a pharmacological approach, we validated the role of RAF1 and BRAF inhibition in enhancing Sorafenib efficacy. AML cell lines were treated with Vemurafenib, GW5074, and AZ628 (which are BRAF, RAF1, and dual BRAF/RAF1 inhibitors, respectively) alone or in combination with Sorafenib. Analyses of cell viability showed minimal effects of Vemurafenib, GW5074, and AZ628 as single agents. By contrast, BRAF or dual BRAF/RAF1 inhibition had additive or synergistic effects with Sorafenib at drug concentrations >1 µM, whereas the RAF1 inhibitor GW5074 increased Sorafenib efficacy only in the OCI-AML3 cell line (Figure S15). These results confirmed that at least down-modulation of BRAF is a mechanism in the enhancement of AML cell sensitivity to Sorafenib.

## 3. Discussion

AML is a rare childhood cancer marked by high biological heterogeneity. One of the most unfavorable patient risk groups is characterized by *MLL* rearrangements. Recently, the methyltransferase DOT1L has emerged as a druggable target for this AML subgroup, since MLL fusions can recruit DOT1L and its mislocation induces aberrant H3K79 methylation and overexpression of leukemia relevant MLL target genes [4,5,7]. Accordingly, the DOT1L inhibitor Pinometostat was evaluated in phase I clinical trials enrolling both pediatric or adult patients with *MLL*-r leukemias. However, it is currently unclear whether DOT1L inhibition may also be therapeutically relevant in the absence of *MLL*-r. In this study, we explored the effects of Pinometostat treatment in both *MLL*-r and non-*MLL*-r AML cells, and we compared the consequences of DOT1L inhibition in these genetically and biologically distinct AML models. We demonstrated that Pinometostat-dependent cytotoxicity is not strictly associated with MLL fusions. For example, anti-proliferative effects were observed in the non-*MLL*-r OCI-AML3 cell line. This is in accordance with previous reports, since OCI-AML3 cells carry *DNMT3A*^R882H^, which is sensitive to DOT1L inhibition [20]. By contrast, the refractoriness to Pinometostat of *MLL*-r THP-1 cells was unexpected. Notably, treatment with Pinometostat had limited effects also in primary *MLL*-r AML cells. This is not surprising, because Pinometostat as a stand-alone therapy achieved limited benefit in clinical trials [16]. However, despite poor cytotoxicity, DOT1L inhibition induced myeloid differentiation and down-modulation of *MEIS1* gene expression in all AML cells irrespectively of *MLL*-r. If, on one hand, these results indicate that cellular factors besides MLL fusions affect DOT1L function, on the other, they suggest that DOT1L plays a critical role in regulating the expression of genes involved in myeloid cell development.

A common feature of AML is the deregulation of signal transduction pathways that are often networked and support the proliferation and survival of leukemic cells, including RAF/MEK/ERK, PI3K/Akt, and the receptor tyrosine kinase FLT3 [33,34,35]. Thus, we decided to investigate these pathways with regard to DOT1L inhibition, observing, however, a limited impact. Global gene expression analysis demonstrated that Pinometostat treatment affected both *MLL*-r and non-*MLL*-r cells, leading to the down-regulation of genes with crucial roles in sustaining leukemic cells, including *MEIS1*, *FLT3*, *TNF,* and *IGF2BP*, and up-regulating genes mainly involved in myeloid differentiation. Notably, it has been reported elsewhere that the critical role of MEIS1 in AML is independent of *MLL*-r [36]. Based on these observations, we hypothesized that DOT1L inhibition could sensitize AML cells to further treatments. It should be considered that there is growing interest in combining anticancer agents with the purpose of increasing therapy benefits, in order to reduce toxicity and overcome the resistance of pediatric AML. We combined Pinometostat with Sorafenib, a multikinase inhibitor which is frequently used to treat poor prognosis pediatric patients with relapsed/refractory AML and *FLT3* mutations [37]. Our results demonstrated that Pinometostat pre-treatment strongly sensitizes AML cell lines and primary cells derived from pediatric AML patients to Sorafenib. Importantly, the combination induced significant cytotoxicity in both *MLL*-r and non-*MLL*-r cells, even if Pinometostat as a stand-alone drug was ineffective. However, we observed no consistent reduction in both FLT3 and its downstream signaling components following combined treatment, suggesting that additional mechanisms account for cell sensitization. Through ChIP-seq analyses of both *MLL*-r and non-*MLL*-r cells, we identified genes directly regulated by DOT1L, and we tried to distinguish among those dependent on MLL fusions. Numerous genes showed high levels of H3K79me2 associated with the presence of MLL fusions, and their methylation is presumably dependent on DOT1L mislocation. However, in these cases, Pinometostat treatment did not necessarily result in gene down-regulation. For example, *CYP1B1*, involved in drug metabolism [38], was H3K79me2 enriched in both *MLL*-r and non-*MLL*-r cells, but it was up-regulated by Pinometostat. By contrast, *IGF2BP2*, whose over-expression correlates with worse overall survival in AML patients [39], and *MEIS1* were H3K79me2 enriched only in *MLL*-r cells, although they were down-regulated by Pinometostat in all AML cells. We also identified DOT1L specific targets commonly methylated in both *MLL*-r and non-*MLL*-r cells, which included numerous genes with prominent roles in cell development, survival, and in leukemogenesis. In particular, in all AML cells, we identified high levels of H3K79me2 in *RAF1* and *BRAF* genes, both encoding for Sorafenib-target kinases, indicating a possible mechanism by which Pinometostat could enhance Sorafenib cytotoxicity. We confirmed that DOT1L regulates expression of RAF1 and BRAF, because Pinometostat treatment affected their expression, and combining Pinometostat and Sorafenib increased BRAF protein down-regulation. Most importantly, BRAF inhibitors also synergized with Sorafenib.

## 4. Material and Methods

### 4.1. Cell Lines and Primary Cultures of AML

WSU-AML cell line was kindly provided by St. Jude Children’s Research Hospital, Memphis, TN, USA. All other AML cell lines were obtained from DSMZ and were cultured in RPMI-1640 (MOLM-13, NOMO-1, THP-1, U-937, HL-60, CMK, KG1a, KASUMI-1, and WSU-AML), IMDM (MV4-11), or MEM-Alpha (OCI-AML3 and HS-5 human stromal cells) media (Thermo Fisher Scientific Inc., Rockford, IL, USA) supplemented with 10%–20% Fetal Bovine Serum (FBS) (Thermo Fisher Scientific Inc.), 100 U/ml penicillin, and 100 μg/ml streptomycin (Sigma-Aldrich, Saint Louis, MO, USA) at 37 °C, in a humidified atmosphere of 5% CO_2_. Samples from pediatric AML patients (n = 15) were collected with informed consent according to institutional guidelines. This study was conducted according to the principals of the Declaration of Helsinki and was approved by the Independent Ethics Committee of the University Hospital of Bologna “S. Orsola-Malpighi” (The approval code: 61/2002/O). Primary AML samples were isolated using Ficoll-Paque (GE Healthcare Bio-Sciences AB, Uppsala, Sweden) and were co-cultured on a layer of human stromal HS-5 cells in DMEM medium supplemented with 20% FBS, penicillin–streptomycin, and human cytokines (100 ng/mL SCF, 10 ng/mL IL3, 20 ng/mL IL6, 10 ng/mL TPO, and 10 ng/mL FLT3L from PeproTech EC, Ltd., London, UK) [18]. Human AML cell lines and pediatric AML primary sample features are summarized in Tables S1 and S2, respectively.

### 4.2. Inhibitors and Treatments

Pinometostat, Sorafenib, Vemurafenib, and AZ628 were purchased from Selleck Chemicals (Houston TX, USA), and GW5074 was purchased from Tocris (Cookson Inc., Avonmouth, UK). All the drugs were dissolved in dimethyl sulfoxide (DMSO) (Sigma-Aldrich) to obtain a 10 mM stock solution. AML cell lines and primary AML cells were cultured for up to 28 days or for 48 h in the presence of Pinometostat or Sorafenib, respectively, or DMSO (control cells). Overall, the final DMSO concentration during all incubations was kept below 0.1%, in order to avoid effects on the differentiation potential of AML cells.

For long-term culture, AML cells and primary samples were plated at a density of 10^5^ and 10^6^ cells/mL, respectively. Growth media and Pinometostat were replaced every 4 days, and, on days of media replacement, leukemic cells were collected, counted, and analyzed as required. For cell viability analysis, the drugs were added at increasing concentrations (from 0.1 to 10 µM Pinometostat; from 1 nM to 10 µM Sorafenib, Vemurafenib, GW5074, and AZ628). For drug-combination experiments with Pinometostat and Sorafenib, both AML cell lines and primary AML samples were first treated with 1 µM Pinometostat for 4 or 8 days, and then 1 µM Sorafenib was added for a further 24 or 48 h (combination times: 4 days + 24 h; 8 days + 24 h; 4 days + 48 h; 8 days + 48 h). For all other analyses, Pinometostat was employed at 1 µM. For drug-combination experiments with Sorafenib, Vemurafenib, GW5074, and AZ628, AML cell lines were plated at a density of 0.2 × 10^6^ cells/mL and treated with single or simultaneously combined agents at increasing concentrations (from 1 nM to 10 µM) for 24 h. 

### 4.3. Statistics

Data are presented as mean values ± SD and were statistically analyzed using a two-tailed unpaired *t*-test or one-way ANOVA with GraphPad Prism Software (GraphPad, San Diego, CA, USA). A *p* value of <0.05 was considered statistically significant.

Additional materials and methods are described in the Supplementary Materials.

## 5. Conclusions

In summary, our data show that DOT1L inhibition has drastic repercussions for the gene expression program in AML cells irrespectively of *MLL*-r, and they provide a comprehensive picture of the cellular functions likely regulated by DOT1L methyltransferase activity. Importantly, our preliminary data on the favorable interaction between Pinometostat and Sorafenib provide the rationale to investigate this drug combination as a novel and more effective approach to treat pediatric AML patients. Although further in vivo analyses are required, our results are of particular interest in light of the limited therapeutic strategies for pediatric AML patients. 

## Figures and Tables

**Table 1:** Selected DOT1L target genes with major roles in cell proliferation and survival.

**Cell division cycle (CDC) genes**
*CDC123 CDC25A CDC27 CDC34 CDC37 CDC40 CDC42 CDC45 CDC6 CDC73 CDCA4 CDCA5 CDCA8*
**Cyclin-dependent kinases**
*CDK1 CDK12 CDK13 CDK4 CDK6 CDK9 CDKN1B CDKN2C*
**Gene involved in transcription regulation**
*E2F1 E2F3 E2F4 ELOF1 ELL EFTUD2 ELP2 EPC1 EPC2 ERH EZH2 EEF2K EEF1B2 EEF1D EEF1E1 EEF1G EEF2 EIF2S2 EIF2A EIF2B4 EIF2B5 EIF2C2 EIF3B EIF3D EIF3E EIF3H EIF3J EIF3L EIF3M EIF4G1 EIF4G3 EIF4A3 EIF4B EIF4E EIF4EBP1 EIF4EBP2 EIF4H EIF5 EIF5A EIF5B EIF6 ETF1*
**Bcl-2 family members**
*BCL2 BNIP2 BAK1 BAG1 BAG5 BAG6 BCLAF1 BCL2L11 BID*
**p53 signaling components**
*TP53RK TP53BP2 MDM2 MDM4*
**Methyltransferases**
*EHMT1 METTL10 METTL11A METTL14 METTL16 METTL19 MLL MLLT1 MLL2 MLL3 MLL5*
**Members of MAP kinases**
*MAPK1 MAPK1IP1L MAPK14 MAPK6 MAPKAP1 MAP2K2 MAP2K3 MAP3K1 MAP3K4 MAP3K7 MAP4K5 MAPKAPK2 MAPKAPK3 MAPKAPK5*
**Cancer related genes**
*PDGFRA MYC PIM3 PARP1 POU2F1 CXCR4 CDKN1B IGF2R*
**Genes encoding for proteins with a reported role in leukemia [27,28,29,30]**
*SETD2 CBFA2T3 CEBPA RUNX1 RUNX2 EZH2 IKZF1 CREB1 FBXW7 NPM1 LMO2 LYL1*
**Genes encoding for proteins with a reported functional interdependence with DOT1L [31,32]**
*BRD4 DUSP6 DOT1L*
**PI3K signaling members**
*AKT2 MCL-1 RICTOR GSK3B PIK3C3*

## Data Availability

Gene expression data have been deposited in the GEO repository (Accession ID: GSE144638). Databank URL: https://www.ncbi.nlm.nih.gov/geo/. ChIP-seq data generated during this study are included in this published article as supplementary tables.

## Supplementary Materials

The following are available online at https://www.mdpi.com/2072-6694/12/7/1972/s1. Supplementary Methods, Figures, and Tables; Table S3: Differentially expressed genes in treated vs. control AML cell lines; Table S4: Genes with a differential H3K79me2 peak enrichment in *MLL*-r compared to non-*MLL*-r AML cell lines; Table S5: Genes with H3K79me2 peak enrichment in both *MLL*-r and non-*MLL*-r AML cell lines.
