# Supplementary material for: Inhibition of Methyltransferase DOT1L Sensitizes to Sorafenib Treatment AML Cells Irrespective of MLL-Rearrangements: A Novel Therapeutic Strategy for Pediatric AML
Source: Cancers (Basel). 2020 Jul 20;12(7):1972. doi: 10.3390/cancers12071972 (PMC7409321; doi:10.3390/cancers12071972)
Supplement: Supplementary file 1 [file cancers-12-01972-s001.zip › cancers-844020-supplementary/Supplementary Methods, Figures and Tables.pdf]

Supplementary Information

Inhibition of Methyltransferase DOT1L Sensitizes to Sorafenib Treatment AML Cells Irrespective of MLL-Rearrangements: A Novel Therapeutic Strategy for Pediatric AML

Annalisa Lonetti [1,*], Valentina Indio [1], Maria Antonella Laginestra [2,3], Giuseppe Tarantino [1], Francesca Chiarini [4,5], Annalisa Astolfi [1,6], Salvatore N. Bertuccio [7], Alberto M. Martelli [8], Franco Locatelli [9], Andrea Pession [1,7,†] and Riccardo Masetti [7,†].

[1] "Giorgio Prodi" Cancer Research Center, University of Bologna, S. Orsola-Malpighi Hospital, Via Massarenti 11, 40138, Bologna, Italy; annalisa.lonetti2@unibo.it (A.L.); valentina.indio2@unibo.it (V.I.); giuseppe.tarantino6@unibo.it (G.T.); annalisa.astolfi@unibo.it (A.A.); andrea.pession@unibo.it (A.P.)

[2] Department of Experimental, Diagnostic, and Specialty Medicine, University of Bologna, S. Orsola-Malpighi Hospital, Via Massarenti 9, 40138, Bologna, Italy; antolaginestra3@gmail.com (M.A.L)

[3] IRCCS Istituto Ortopedico Rizzoli, Laboratory of Experimental Oncology, via di Barbiano 1/10 40136 Bologna Italy

[4] Institute of Molecular Genetics, Luigi Luca Cavalli-Sforza — CNR National Research Council of Italy, 40136, Bologna, Italy; francesca.chiarini@gmail.com (F.C.)

[5] IRCCS Istituto Ortopedico Rizzoli, via di Barbiano 1/10 40136,, Bologna, Italy

[6] Department of Morphology, Surgery and Experimental Medicine, University of Ferrara, Via Luigi Borsari 46, 44121 Ferrara, Italy; annalisa.astolfi@unife.it (A.A.)

[7] Pediatric Hematology-Oncology Unit, Department of Medical and Surgical Sciences DIMEC, University of Bologna, S. Orsola-Malpighi Hospital, Via Massarenti 11, 40138, , Bologna, Italy; salvatore.bertuccio2@unibo.it (S.N.B.); andrea.pession@unibo.it (A.P.); riccardo.masetti5@unibo.it (R.M.)

[8] Department of Biomedical and Neuromotor Sciences, University of Bologna, Via Irnerio 48, 40126, Bologna, Italy; alberto.martelli@unibo.it (A.M.M.)

[9] Department of Pediatric Hematology-Oncology and Cell and Gene Therapy, IRCCS Ospedale Pediatrico Bambino Gesù, Sapienza University of Rome, Piazza Sant'Onofrio 4, 00165 Rome, Italy; franco.locatelli@opbg.net (F.L.)

* Correspondence: annalisa.lonetti2@unibo.it

† The last two authors contributed equally to this work.

Supplementary Materials and Methods

Cell viability analysis

Concentration- and time-dependent effects on cell viability of Pinometostat (as single agent or in combination with Sorafenib) were determined through flow cytometry absolute count of viable cells, employing the 123count eBeads and Propidium iodide staining (Thermo Fisher Scientific Inc., Rockford, IL, USA) to identify death cells. Cell proliferation following treatment with the multi-kinase inhibitor Sorafenib, the BRAF inhibitor Vemurafenib, the RAF1 inhibitor GW5074 and the dual BRAF/RAF1 inhibitor AZ628 was assessed using the MTT [3-(4,5-Dimethylthythiazol-2-yl)-2,5-diphenyltetrazolium bromide] cell proliferation kit (Roche Diagnostic, Basel, Switzerland), according to manufacturer's instructions. IC_{50} and CI (combination index) values were determined using the CompuSyn software (1). Experiments were repeated at least two times, and data were represented as the mean ± SD.

Induction of cell differentiation

To induce myeloid-monocytic differentiation, AML cell lines were cultured in the appropriate medium in the presence of phorbol 12-myristate 13-acetate (PMA) (Sigma-Aldrich, Saint Louis, MO, USA). A concentration of 100 ng/mL of PMA was employed for MV4-11, MOLM-13, OCI-AML3 and U-937 cells, whereas NOMO-1, THP-1 and HL-60 cells were treated with 20 ng/mL of PMA. Following 48 hours incubation, the morphology of the cells was assessed under a microscope and adherent PMA-differentiated cells were used as required.

Annexin V-FITC/PI staining and cell cycle analysis

Apoptosis and cell cycle analysis were performed as previously described (2). AML cell lines and primary AML cells were treated with Pinometostat and Sorafenib (alone or in combination) or the vehicle alone (DMSO 0.01%) for up to 28 or 16 days, respectively. Analyses were performed on an FC500 flow cytometer (Beckman Coulter, Brea, CA, USA) with the appropriate software (CXP, Beckman Coulter). At least 10,000 events per sample were acquired.

Flow Cytometry analysis of surface markers

To characterize the immunophenotipic differentiation induced by Pinometostat, AML cell lines were treated with the drug or the vehicle alone (DMSO 0.01%) and harvested every 4 days until 28 days to measure the expression of CD11b and CD14 surface markers. The cells were washed with PBS and labeled with PE-conjugated anti-human CD11b or CD14 antibody, or PE-labeled IgG isotype control (BD Biosciences, Franklin Lakes, NJ, USA). For analysis of FLT3 expression, untreated cells were incubated with PE-conjugated anti-human CD135 antibody (BD Biosciences). Data were acquired on an FC500 flow cytometer (Beckman Coulter) and analyses were performed

with the appropriate software (CXP, Beckman Coulter). At least 10,000 events per sample were acquired.

Protein Extraction and Western Blot Analysis

To analyze inhibition of H3K79 methylation and resultant pathway modulation, cells were incubated for up to 28 days with Pinometostat and Sorafenib (alone or in combination) or the vehicle alone (DMSO 0.01%) and collected by centrifugation every 4 days. Histones were extracted by overnight acid extraction using the protocol outlined on the Abcam website (www.abcam.com) and whole-cell protein extracts were prepared using the M-PER Mammalian Protein Extraction Reagent supplemented with the Protease and Phosphatase Inhibitor Cocktail (Thermo Fisher Scientific Inc.). Immunoblotting was performed using standard procedures as previously described (3). 30 µg of proteins or 5 µg of histones were separated by SDS– PAGE using 4-20% Criterion TGX polyacrylamide gels (Bio-Rad, Hercules, CA, USA) and blotted onto a nitrocellulose membrane (Bio-Rad). All primary and secondary antibodies were from Cell Signaling Technology. Proteins were detected using the Amersham ECL Prime Western Blotting Detection Reagent (GE Healthcare, Little Chalfont, Buckinghamshire, England), the ChemiDoc-It2 Imaging System and the VisionWorksLS Software for the analysis (UVP, LLC, Upland, CA).

RT-PCR analysis

RNA extraction, reverse transcription and Real-Time (RT) PCR analysis were performed as previously reported (4). AML cell lines and primary AML cells were treated with Pinometostat for up to 16 or 8 days, respectively. Expression of *HOXA9*, *MEIS1*, *FLT3*, *STAT5a*, *c-MYC, RAF1* and *BRAF* genes was assessed using commercially available TaqMan assays (Applied Biosystems, Foster City, CA, USA) on a 7300 real-time PCR system (Applied Biosystems). RNA 18S ribosomal 1 (Applied Biosystems) was used as control gene and the Stratagene's Universal Human Reference RNA was used as calibrator (Stratagene, La Jolla, CA). Results were expressed as $2^{-\Delta Ct}$ to compare the relative gene expression among samples, or as $2^{-\Delta\Delta Ct}$ to compare gene expression of the treated cells compared to untreated controls (5).

Gene expression microarray analysis

Total RNA was isolated from AML cell lines treated for 8 days with 1 µM Pinometostat or vehicle alone (DMSO 0.01%) using the RNeasy Mini Kit (Qiagen, Venlo, The Netherlands) according to the manufacturer's instructions. Gene expression profile was assessed in duplicate using the human Clariom™ S Assays (Thermo Fisher Scientific) and samples were prepared and hybridized according to the manufacturer's recommendations. All calculations were performed using R version 3.6.1 (Bioconductor). CEL files raw data were normalized using Robust Multi-Array Average normalization (RMA), log2 transformed and annotated by pd.clariom.s.human package.

Differentially expressed genes were computed by Linear expression models using the limma bioconductor package (6). Principal component analysis (PCA) was carried out using the R package prcomp. Supervised analysis was used to compare within each cell line the conditions treatment *vs* control. The identified differentially expressed genes were filtered basing on p value (<0.05) and fold change (absolute FC > 1) and compared between cell lines. Genes with a concordant up- or down-modulation in at least three cell lines were further selected (n=171). The functional enrichment analysis web tool WebGestalt (WEB-based Gene SeT AnaLysis Toolkit) was used for the identification of signaling pathways. The HeatMap showing the fold change value of the 171 selected genes was carried out by R–bioconductor Complex Heatmap package (clustering method: ward.D, clustering distance: manhattan). Gene expression data have been deposited into the GEO database (GEO accession number GSE144638).

Chromatin Immunoprecipitation (ChIP) sequencing (ChIP-seq) and Data Analysis

Samples preparation. ChIP was performed employing the iDeal ChIP-seq kit for Histones (Diagenode, Denville, NJ) using the provided lysis, dilution, and wash buffers and according to manufacturer's protocol. Crosslinking was performed with 1% formaldehyde for 10 min, and the reaction was stopped by the addition of glycine. The fixed cells were lysed in the provided lysis buffers, and chromatin was fragmented by sonication on a Bioruptor sonicator (Diagenode) in a constantly circulating 4°C water bath. Sheared chromatin was analyzed on a 1.5% agarose gel to assess optimal range fragmentation (200-500 bp). For each sample, 1 µl of sheared chromatin was placed aside to be subsequently used as input. For ChIP, the sheared chromatin was incubated with a H3K79me2 specific antibody (Cell Signaling Technology) and collected with magnetic beads. Eluted DNA fragments were purified by using a Qiagen PCR purification kit (Qiagen, Valencia, CA), quantified with the fluorimetric QuantIT Picogreen assay (Thermo Fisher Scientific) and directly subjected to qPCR or high-throughput sequencing.

ChIP qPCR. ChIP samples were quantified relative to inputs as elsewhere reported (7). The amount of genomic DNA co-precipitated with antibody was calculated as a percentage of total input using the following formula: $\Delta CT = CT\ (input) - CT\ (ChIP)$. Total recovery percentage was calculated as $2^{\Delta CT} \times 1.0\%$ (as 1 µl out of 100 µl sheared chromatin was used as input). Quantitative real-time PCR (qPCR) of ChIPed DNA was performed using SYBR™ Green PCR Master Mix (Thermo Fisher Scientific) and Light Cycler 480 system (Roche Diagnostics, Rotkreuz, Switzerland). Primer sequences are listed in Supplementary Table S6.

ChIP-Seq. Libraries were synthesized employing the iDeal Library Preparation kit (Diagenode), according to manufacturer's instructions. ChIPed DNA/inputs and libraries were quantified using the fluorimetric QuantIT Picogreen assay (Thermo Fisher Scientific). Libraries were further quality-checked and sized with Bioanalyzer 2100 using DNA High Sensitivity chip (Agilent Technologies, Santa Clara, CA, US). Samples were sequenced at 79X2 paired-end mode on a NextSeq500

instrument (Illumina, San Diego, CA). ChiP-Seq data analysis was performed on server Centos 5 implementing a custom open source bioinformatic pipeline. Firstly, the paired-end reads were trimmed to remove sequence adapters and low quality bases with AdapterRemoval v.1.5.4 (8). Then, the cleaned reads were mapped on reference human genome hg19 with Bowtie2 v.2.2.5 (9) and only the unique-mapped-reads were kept and considered for the following analysis. Peak calling was performed using MACS2 v.2.1.1 (10) adopting "callpeak" function (with default parameters) implemented to normalize each sample against a input track, to call the immunoprecipitated regions and to quantify the fold-change enrichment of each region called. Subsequently, BAM alignments and MACS2 results were analyzed with the aims to define the set of genomic regions differentially and commonly immunoprecipitated between *MLL*-r and non *MLL*-r cells. In details, the differential binding analysis was performed with several methods all implemented in R-bioconductor (www.bioconductor.org) including csaw, DESeq2, edgeR and limma. Where necessary, the reads raw count was performed with the Phyton package HTseq-count (https://htseq.readthedocs.io). A stringent consensus method was then adopted to combine the obtained results; in particular, we defined as "high confidence differentially bound sites" the genomic regions called with significant differences (*p*-value<0.05) in all the four packages adopted. Differently, the commonly bound sites were selected from MACS2 results by intersecting (bedtools intersect - https://bedtools.readthedocs.io) all the regions with fold-change enrichment > 10 and q-value<10-3. The genomic regions were annotated with the corresponding gene symbol and the bp distance from transcription start site with the tool Homer (http:// http://homer.ucsd.edu). Gene Ontology categorization was performed with the tool CateGOrizer (http://ssbio.cau.ac.kr/software/categorizer/) and relevant cancer genes were identified by comparison with the Cancer Gene Census (version 89). Coverage for aggregate profile plots and for each gene plot was normalized using the total number of mapped reads of the sample and displayed using the R library CoverageView and Plotly, respectively.

Supplementary Figures

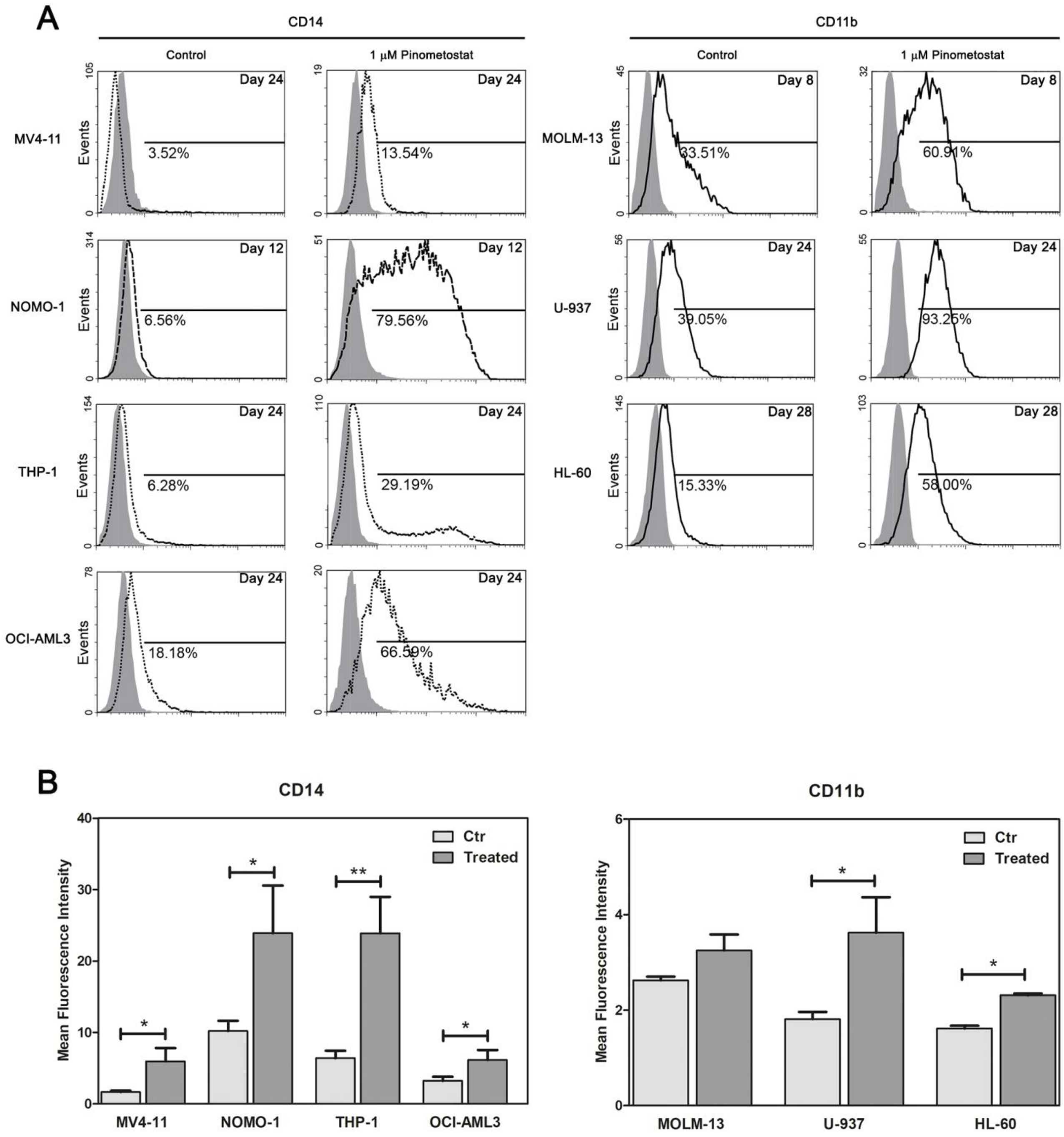

Figure S1: a Histograms relative to flow cytometry analysis and showing an increase in CD11b or CD14 expression in AML cell lines following treatment with 1 µM Pinometostat. A representative time points for each cell line is shown. Control cells were treated with vehicle alone (DMSO 0.01%). **b** Mean fluorescence intensity (mfi) of CD14 or CD11b AML cells quantified every 4 days and up to day 28 in both control and 1 µM Pinometostat-treated AML cell lines. Data are represented as mean ± SD. Asterisks indicate levels of significance (* $p \leq 0.05$, ** $p \leq 0.01$).

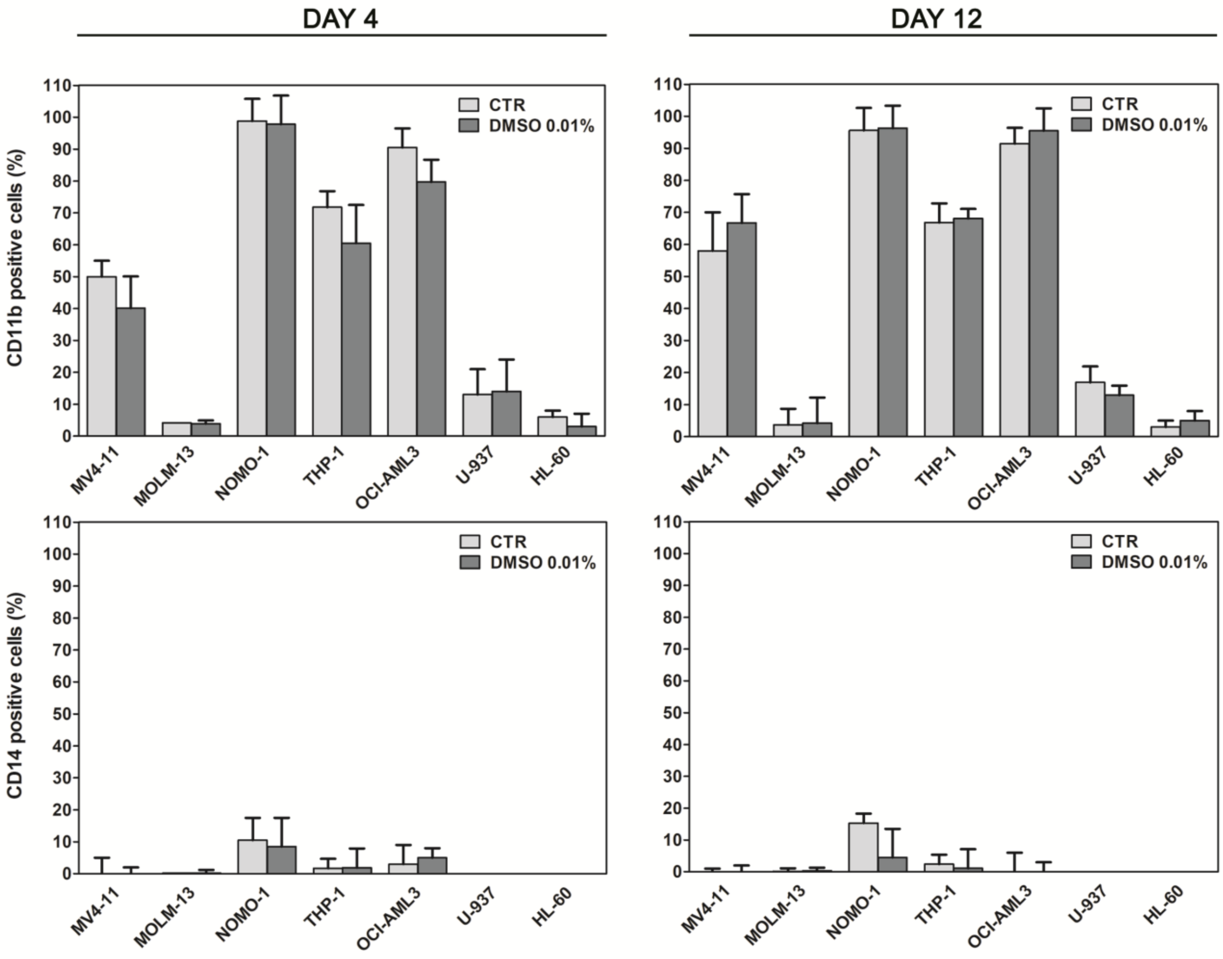

Figure S2: Assessment of DMSO effects on cell differentiation. AML cell lines were cultured up to 12 days with or without DMSO 0.01% and were tested by flow cytometry to assess the expression of CD11b and CD14 surface markers. No significant differences were observed following DMSO long-term exposure between untreated and DMSO-treated cells, demonstrating that such DMSO concentration did not induce cell differentiation. Results of three independent replicates are presented as means ± SD.

A

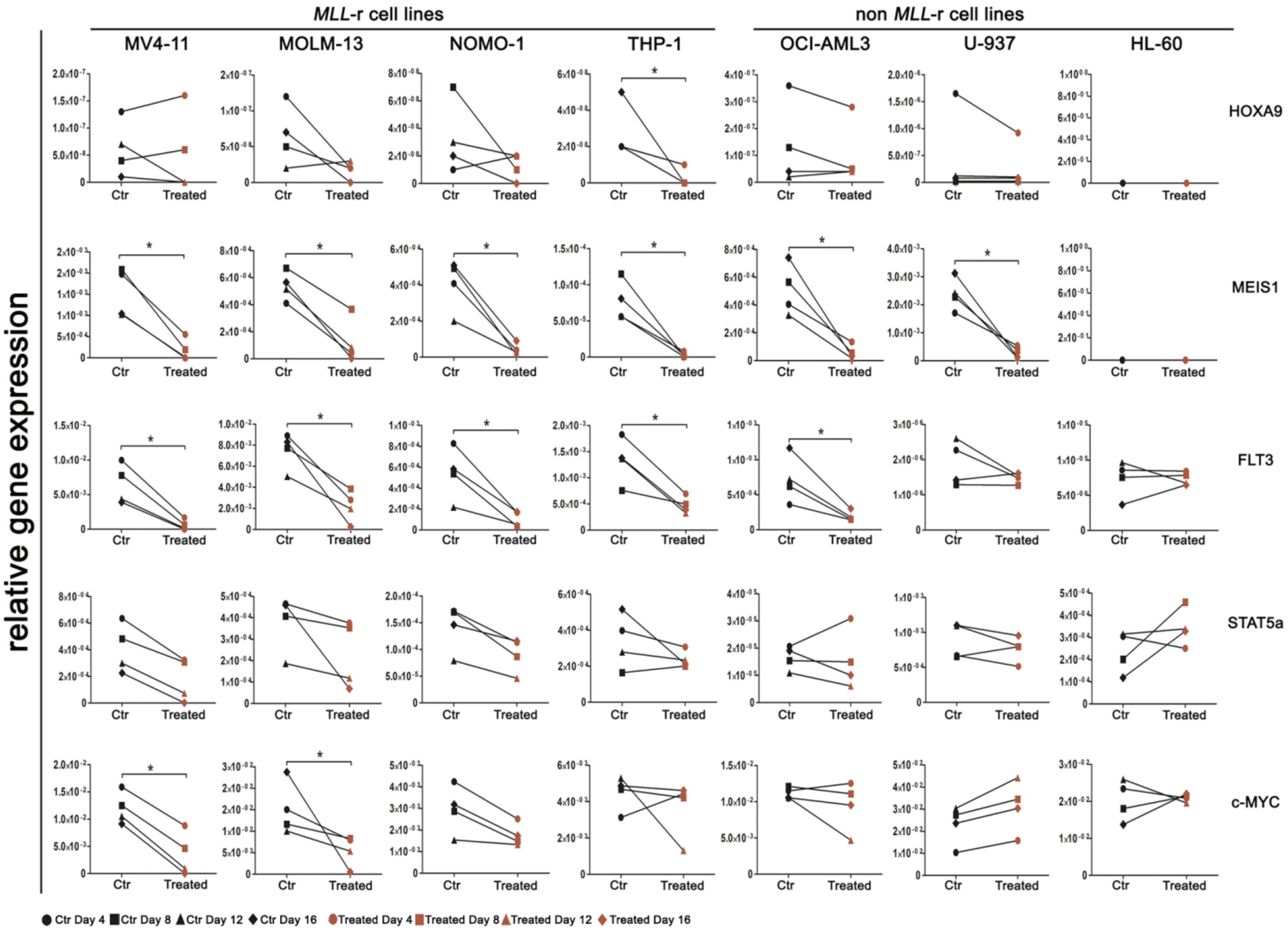

MLL-r cell lines
non *MLL*-r cell lines
MV4-11
MOLM-13
NOMO-1
THP-1
OCI-AML3
U-937
HL-60
relative gene expression
HOXA9
MEIS1
FLT3
STAT5a
c-MYC
Ctr
Treated
*
Ctr Day 4
Ctr Day 8
Ctr Day 12
Ctr Day 16
Treated Day 4
Treated Day 8
Treated Day 12
Treated Day 16

B

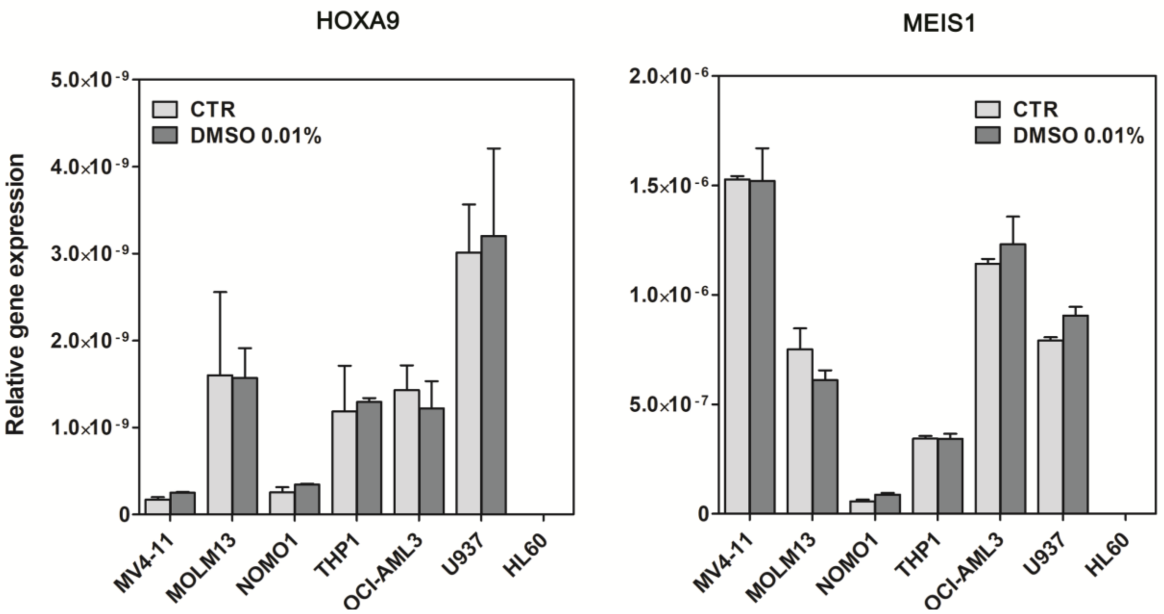

Figure S3: a Gene expression analysis of components that are crucial for proliferation and survival. AML cell lines, exposed to Pinometostat for 16 days, and control cells (DMSO 0.01%) were tested by RT-PCR every 4 days to assess drug-induced changes in gene expression (* $p<0.05$). **b** Assessment of DMSO effects on *HOXA9* and *MEIS1* gene expression. AML cell lines were exposed to DMSO 0.01% and *HOXA9* and *MEIS1* mRNA levels were compared to that of control cells. A representative experiment showing relative gene expression after 4 days exposure to DMSO 0.01% is shown. CTR: control (untreated cells). Results of three independent replicates are presented and data are expressed as $2^{\Delta Ct}$±SD.

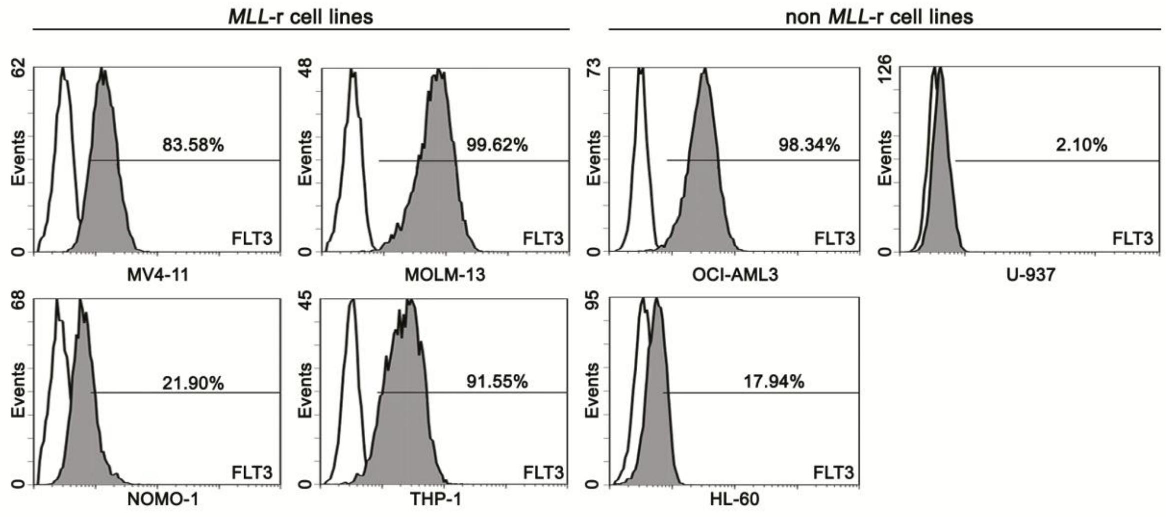

Figure S4: Flow cytometry analysis of FLT3 expression in human AML cell lines employed in the present study.

A

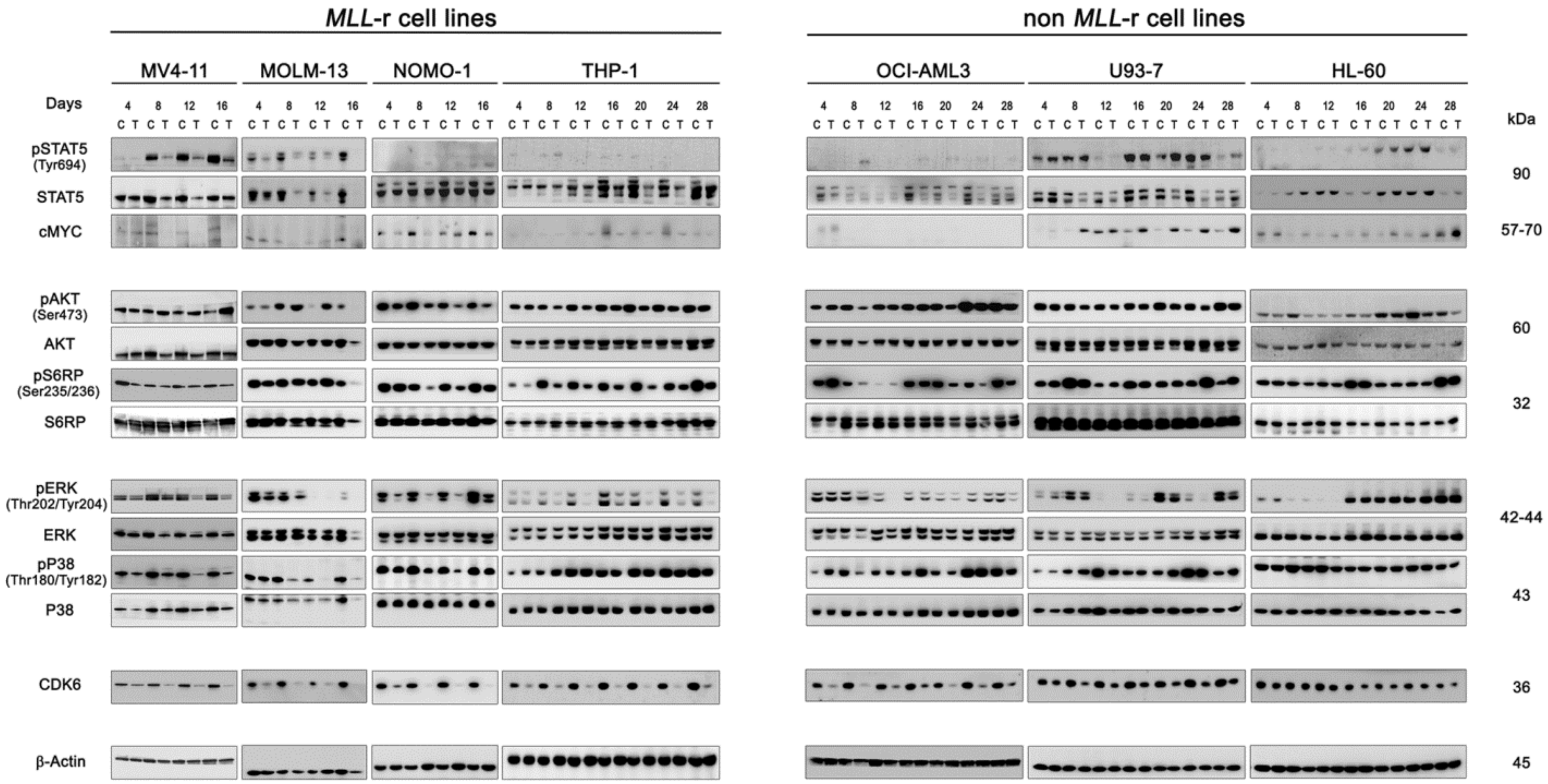
MLL-r cell lines
non MLL-r cell lines
MV4-11
MOLM-13
NOMO-1
THP-1
OCI-AML3
U93-7
HL-60
Days
4 8 12 16
4 8 12 16 20 24 28
C T
kDa
pSTAT5 (Tyr694)
STAT5
cMYC
pAKT (Ser473)
AKT
pS6RP (Ser235/236)
S6RP
pERK (Thr202/Tyr204)
ERK
pP38 (Thr180/Tyr182)
P38
CDK6
β-Actin
90
57-70
60
32
42-44
43
36
45

B

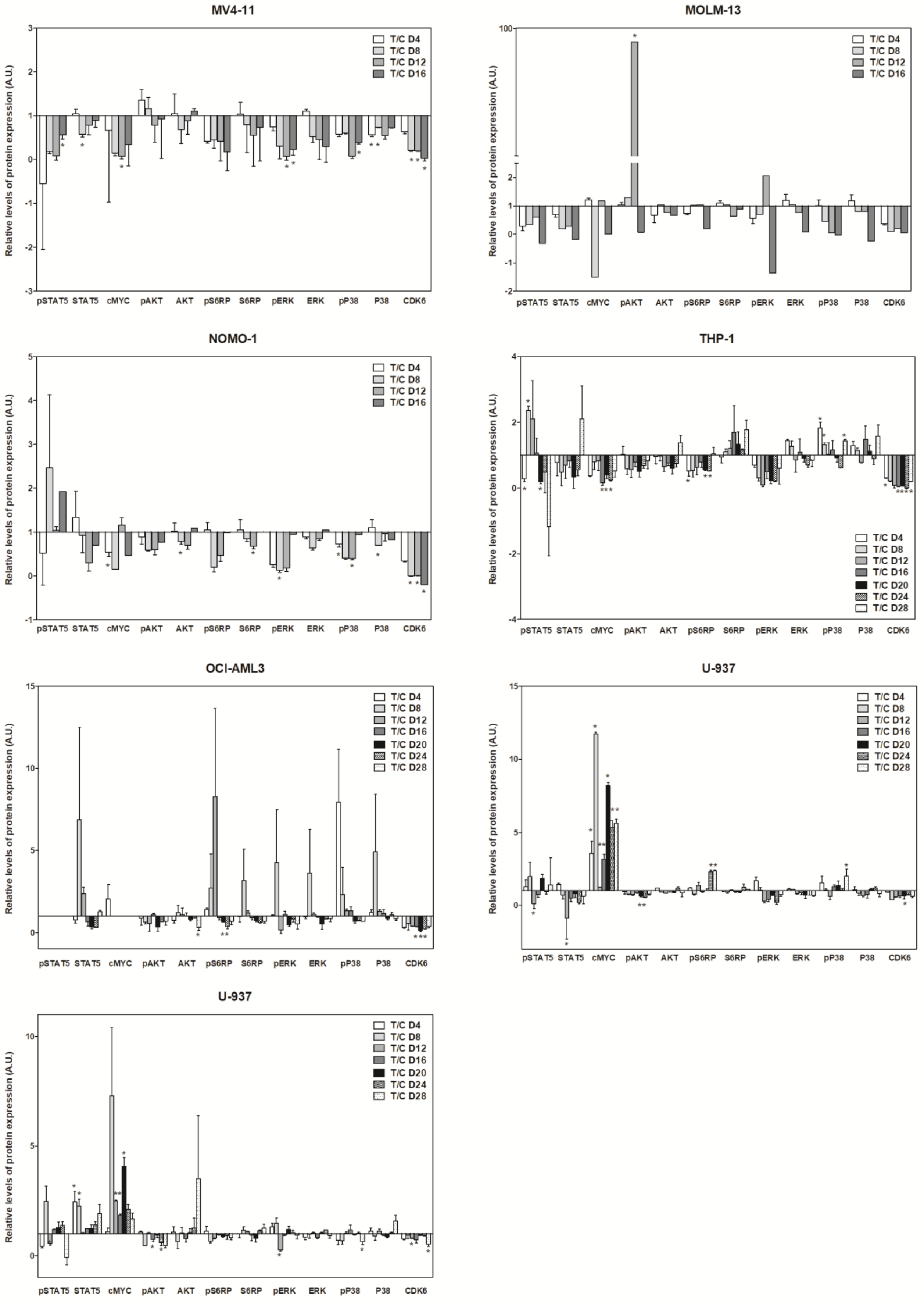
MV4-11
MOLM-13
NOMO-1
THP-1
OCI-AML3
U-937
U-937
Relative levels of protein expression (A.U.)
pSTAT5 STAT5 cMYC pAKT AKT pS6RP S6RP pERK ERK pP38 P38 CDK6
T/C D4
T/C D8
T/C D12
T/C D16
T/C D20
T/C D24
T/C D28

Figure S5: Impact of Pinometostat treatment on FLT3, PI3K/Akt and MEK/ERK pathways. **a** *MLL*-r and non *MLL*-r AML cell lines were cultured for up to 28 days in the presence of 1 µM Pinometostat or 0.01% DMSO (control cells), and western blot analysis was then performed every 4 days, as indicated. Antibody to β-Actin served as a loading control. Molecular weights are indicated at right. C: control cells; T: treated cells. **b** Densitometric analysis of western blots. Data were normalized on β-Actin and represented as the ratio between relative protein expression in treated *vs* control cells at each time point. Results represent mean values ± SD (* $p \leq 0.05$).

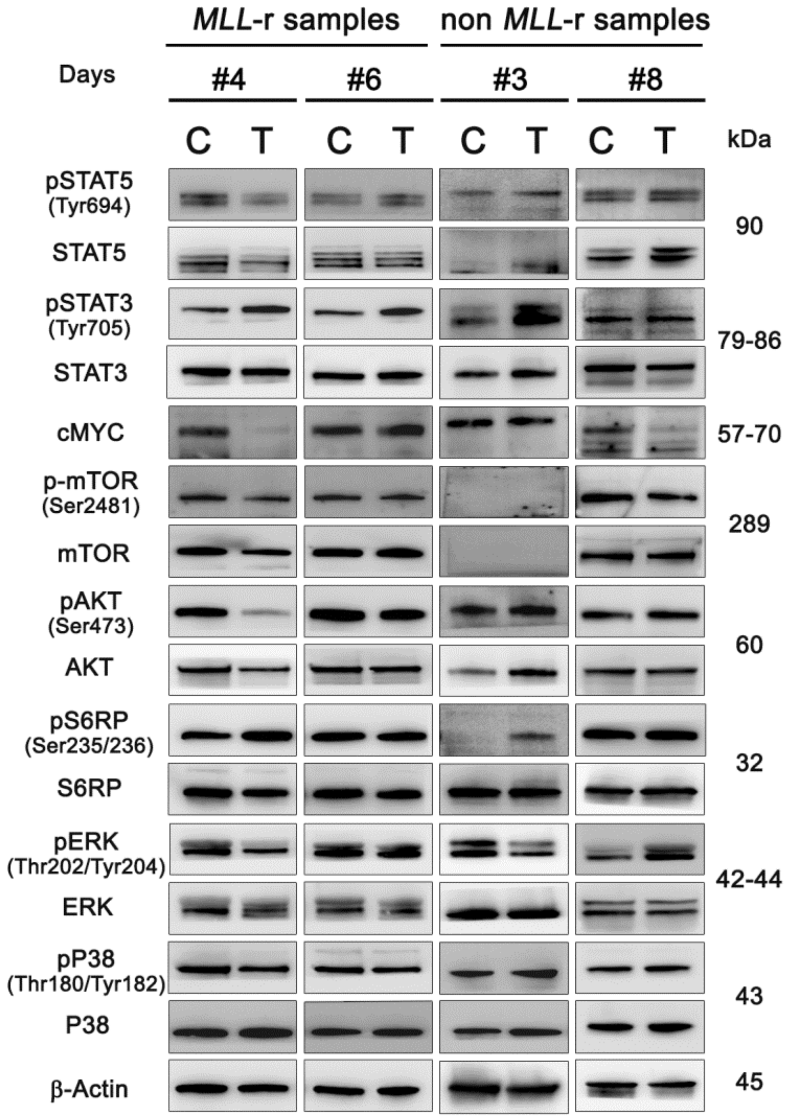

Figure S6: Impact of Pinometostat treatment on FLT3, PI3K/Akt and MEK/ERK pathways in primary AML samples. Primary cells were cultured for 8 days in the presence of 1 µM of Pinometostat or 0.01% DMSO (control cells), and western blot analysis was then performed. Antibody to β-Actin served as a loading control. Molecular weights are indicated at right. C: control cells; T: treated cells.

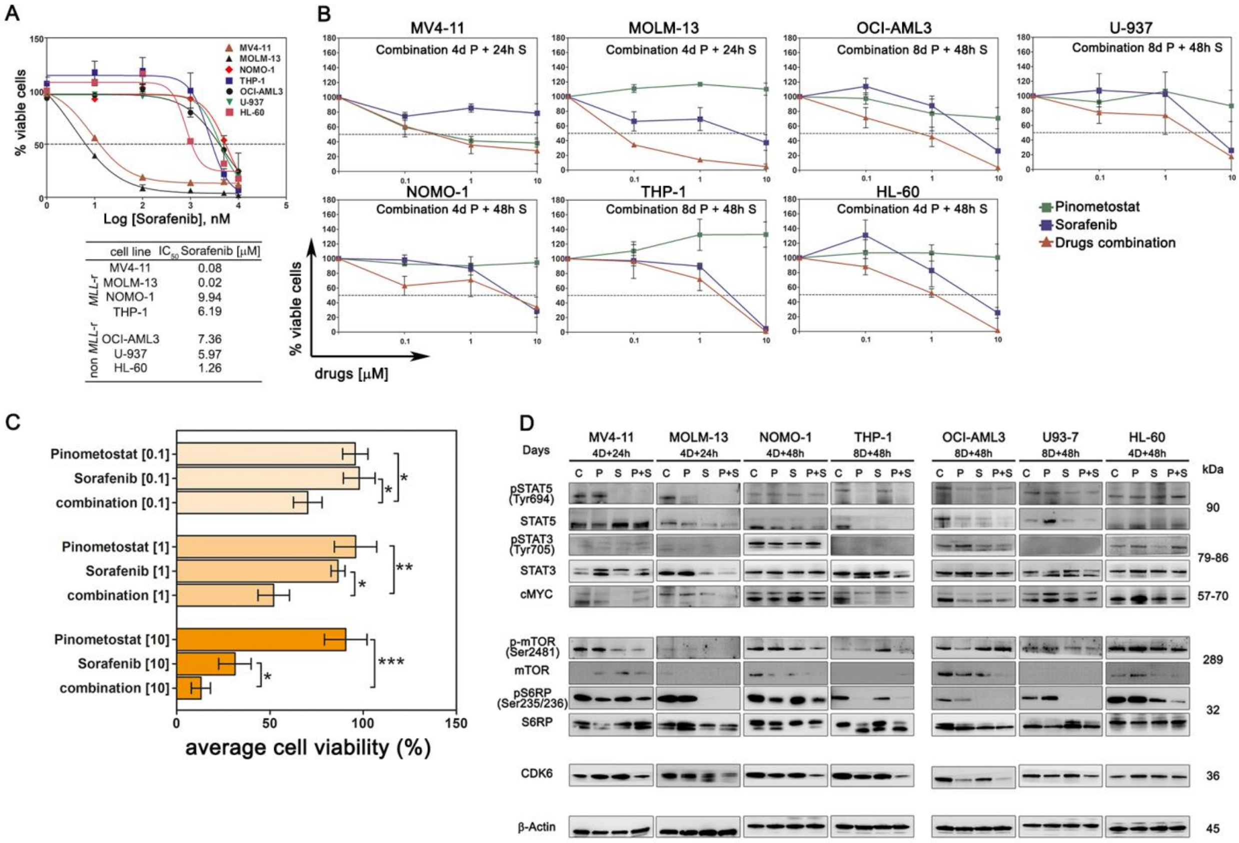

Figure S7: Pinometostat pre-treatment sensitize human AML cell lines to Sorafenib. **a** Growth curves of *MLL*-r and non *MLL*-r AML cell lines treated with increasing concentration of Sorafenib for 48 hours and summary of IC_{50} concentrations. **b** Growth curves of *MLL*-r and non *MLL*-r AML cell lines pre-treated with Pinometostat before Sorafenib addition (Pinometostat/Sorafenib ratio 1:1). Combination times are indicated in each panel (4/8d: 4/8 days; 24/48h: 24/48 hours). In **a** and **b** results of three independent replicates are presented as means ± SD. **c** Comparison of the effects on cell viability induced by Pinometostat and Sorafenib as single agents or in combination at 0.1, 1 and 10 μM. Each bar represents the mean percentage of viable cells among all AML cell lines at the time points indicated in figure S7b, and data are presented as means ± SD. **d** Western blot analysis of FLT3, PI3K/Akt and MEK/ERK pathways in AML cell lines treated with Pinometostat and Sorafenib as single agents or in combination. Combination times are indicated for each cell line. Antibody to β-Actin served as a loading control. Molecular weights are indicated at right. C: control cells; P: Pinometostat treatment; S: Sorafenib treatment; P+S: combined treatment (Pinometostat and Sorafenib).

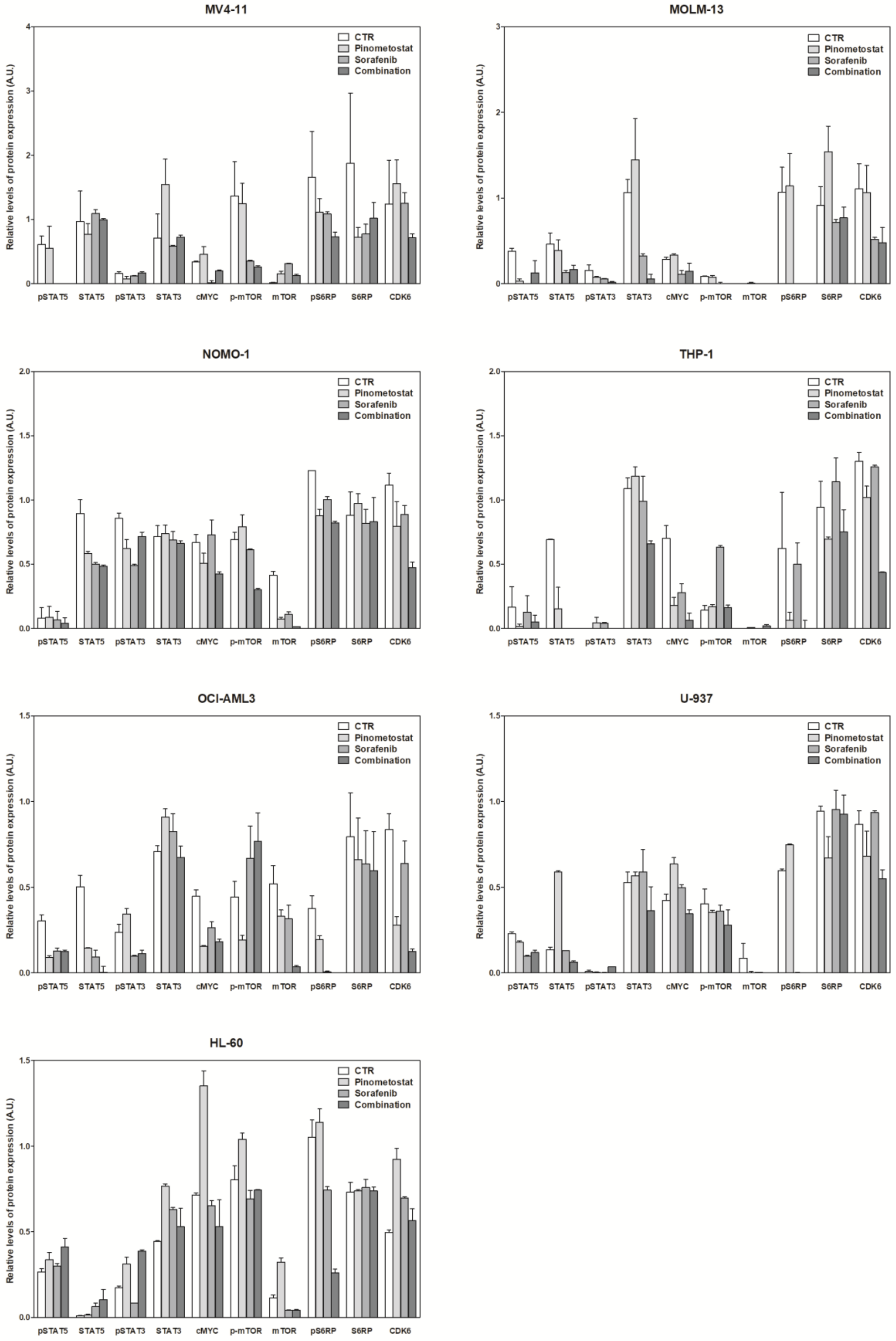

Figure S8: Densitometric analysis of western blots shown in Figure S7. Data were normalized on β-Actin. Results represent mean values ± SD.

.

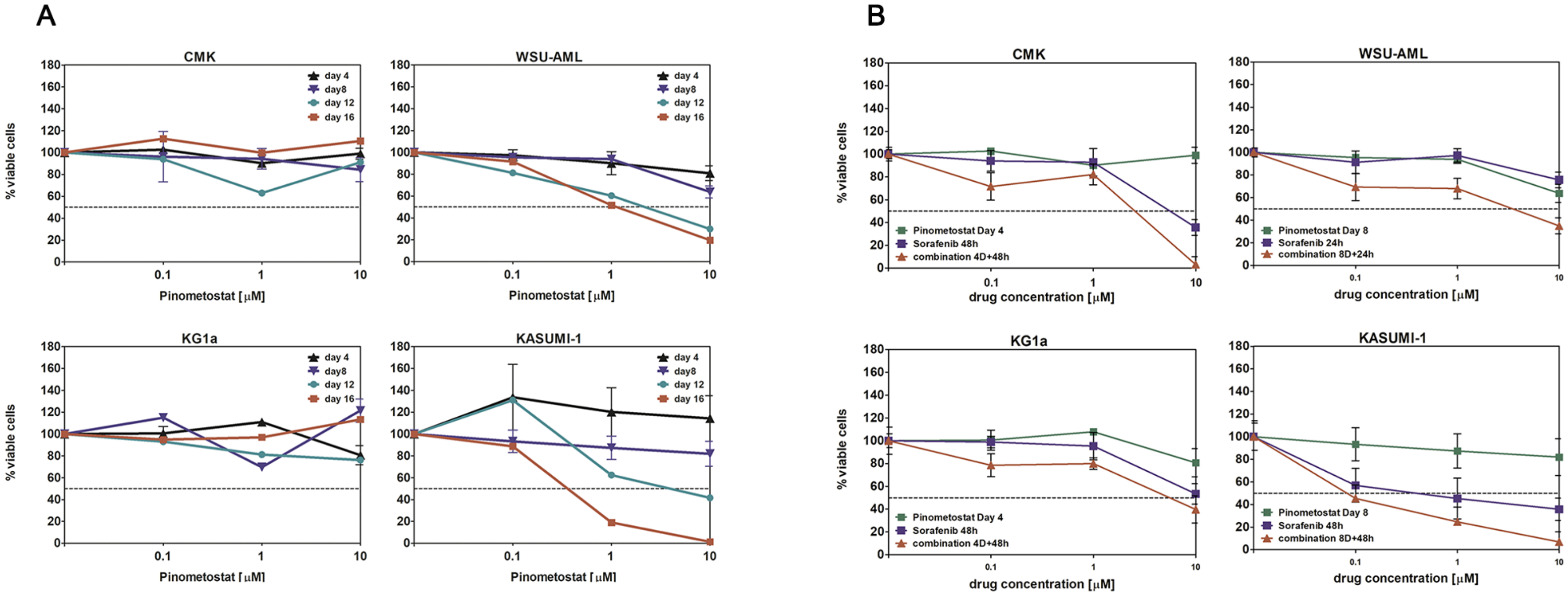

Figure S9: Validation of enhancement of Sorafenib efficacy induced by Pinometostat in AML cell lines with wild type *MLL*. **a** Growth curves of additional non *MLL*-r AML cell lines treated with increasing concentrations of Pinometostat for up to 16 days. In spite the absence of MLL fusions, KASUMI-1 cell line displayed sensitivity to Pinometostat as single agent. **b** Growth curves of non *MLL*-r AML cell lines pre-treated with Pinometostat before Sorafenib addition (Pinometostat/Sorafenib constant ratio 1:1). Combination times are indicated in each panel. Results of three independent replicates are presented as means ± SD.

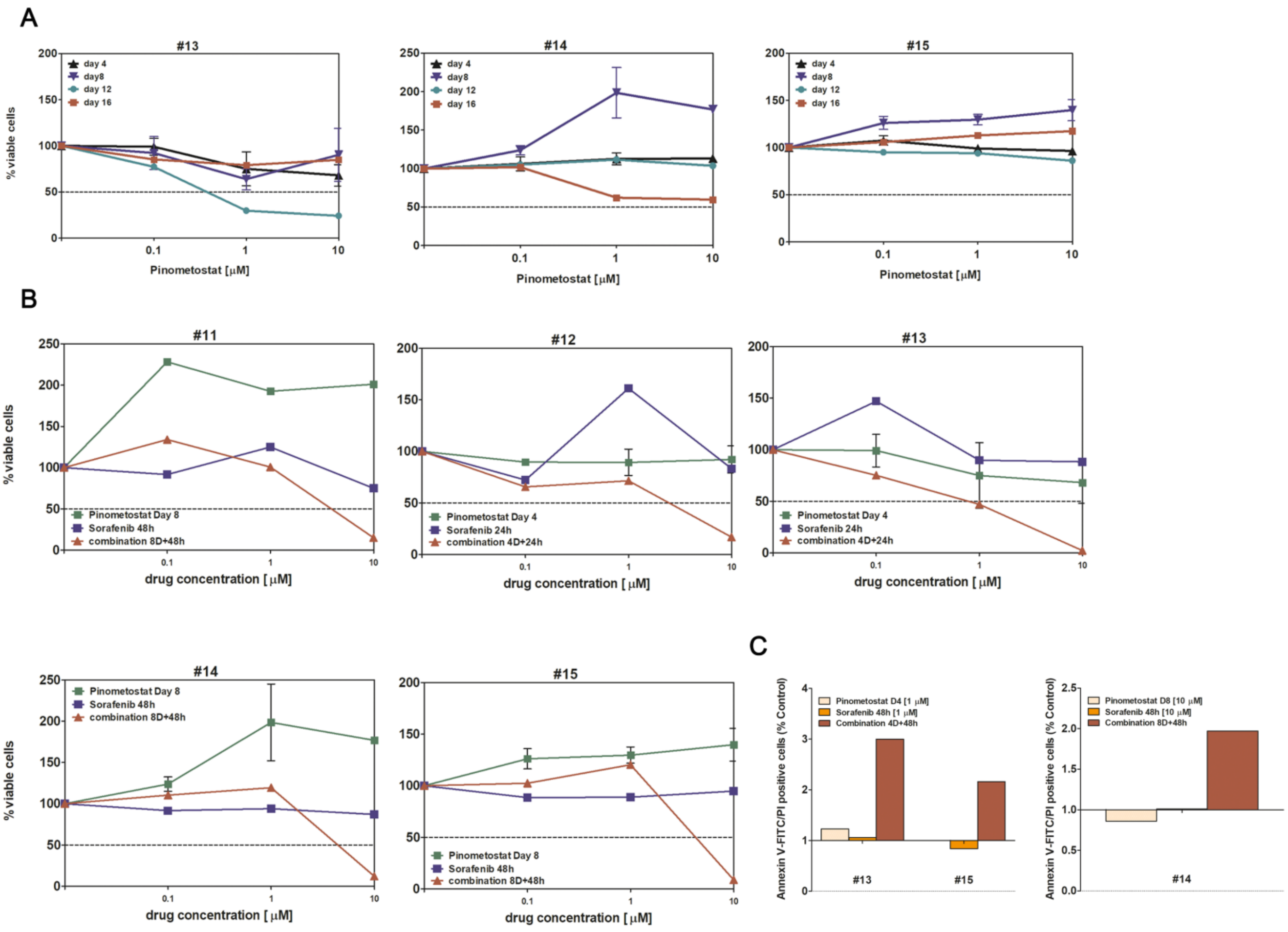

Figure S10: Analysis of additional non *MLL*-r primary samples confirmed the efficacy of combined treatment in this genetic subgroup of AML samples. **a** Effects of Pinometostat as single agent. AML primary samples were treated with increasing concentrations of Pinometostat for up to 16 days, showing almost no effects of the drug. **b** Effects of the combination in the additional subset of non *MLL*-r primary samples. Combination times are indicated in each panel. In **a** and **b** results of three independent replicates are presented as means ± SD. **c** Flow cytometry detection of apoptosis in AML primary samples following combined treatment according to the scheme of pre-treatment with Pinometostat. Data are expressed as percentage of apoptotic cells relative to control, and show an increase of apoptotic/necrotic cells induced by the drug combination.

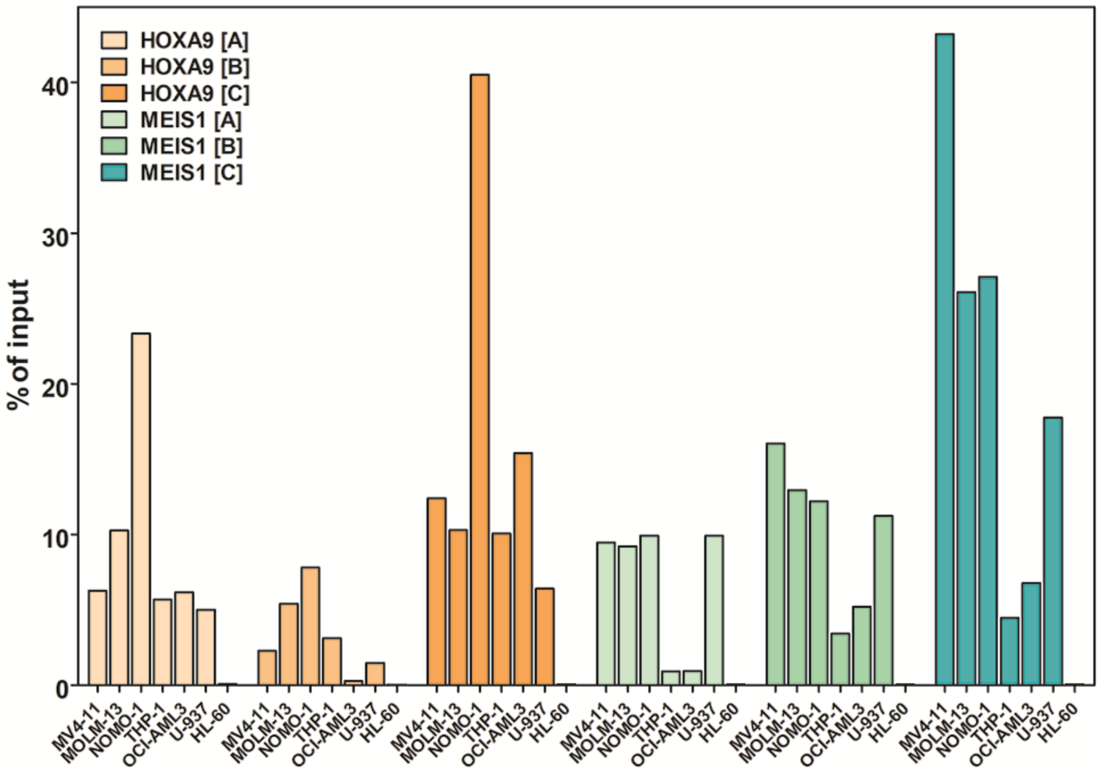

Figure S11: qPCR analysis, following ChIP with a H3K79me2 specific antibody, showing H3K79me2 enrichment of *HOXA9* and *MEIS1* genes. qPCR analysis was performed employing different primers located upstream *HOXA9* and *MEIS1* transcription start site, TSS (HOXA9 [A] and MEIS1 [A]); at TSS (HOXA9 [B] and MEIS1 [B]); downstream the TSS (HOXA9 [C] and MEIS1 [C]).

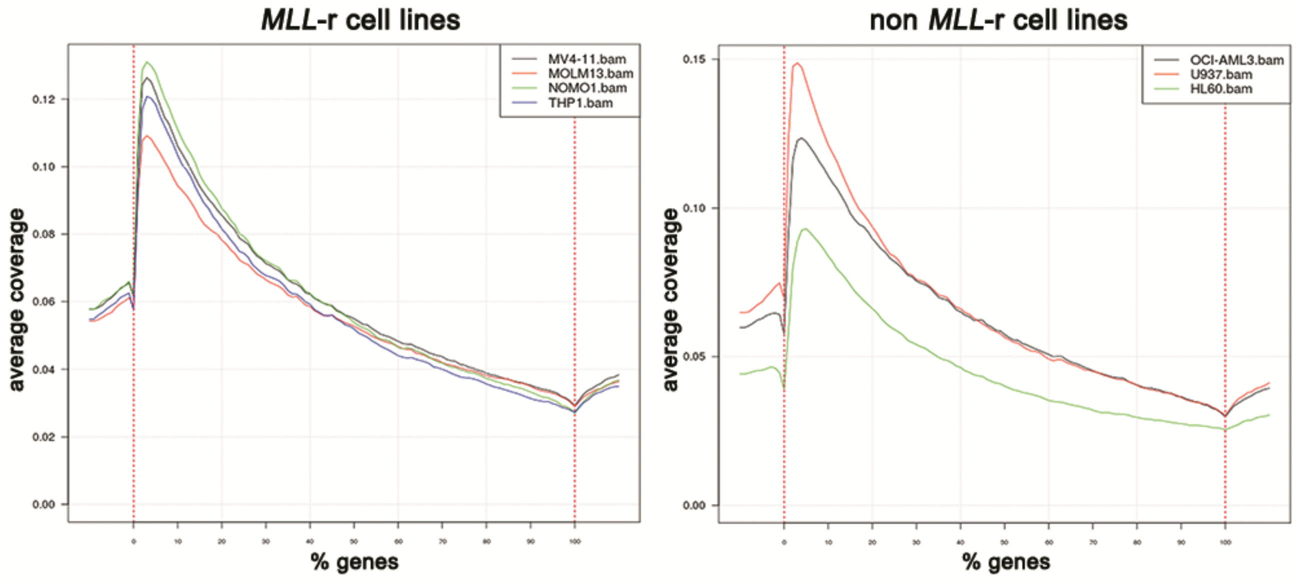

Figure S12: Analysis of ChIP-seq data from human AML cell lines. Overlay plots showing an averaged profile of H3K79me2 level across the genome in *MLL*-r (left) and non *MLL*-r (right) cell lines.

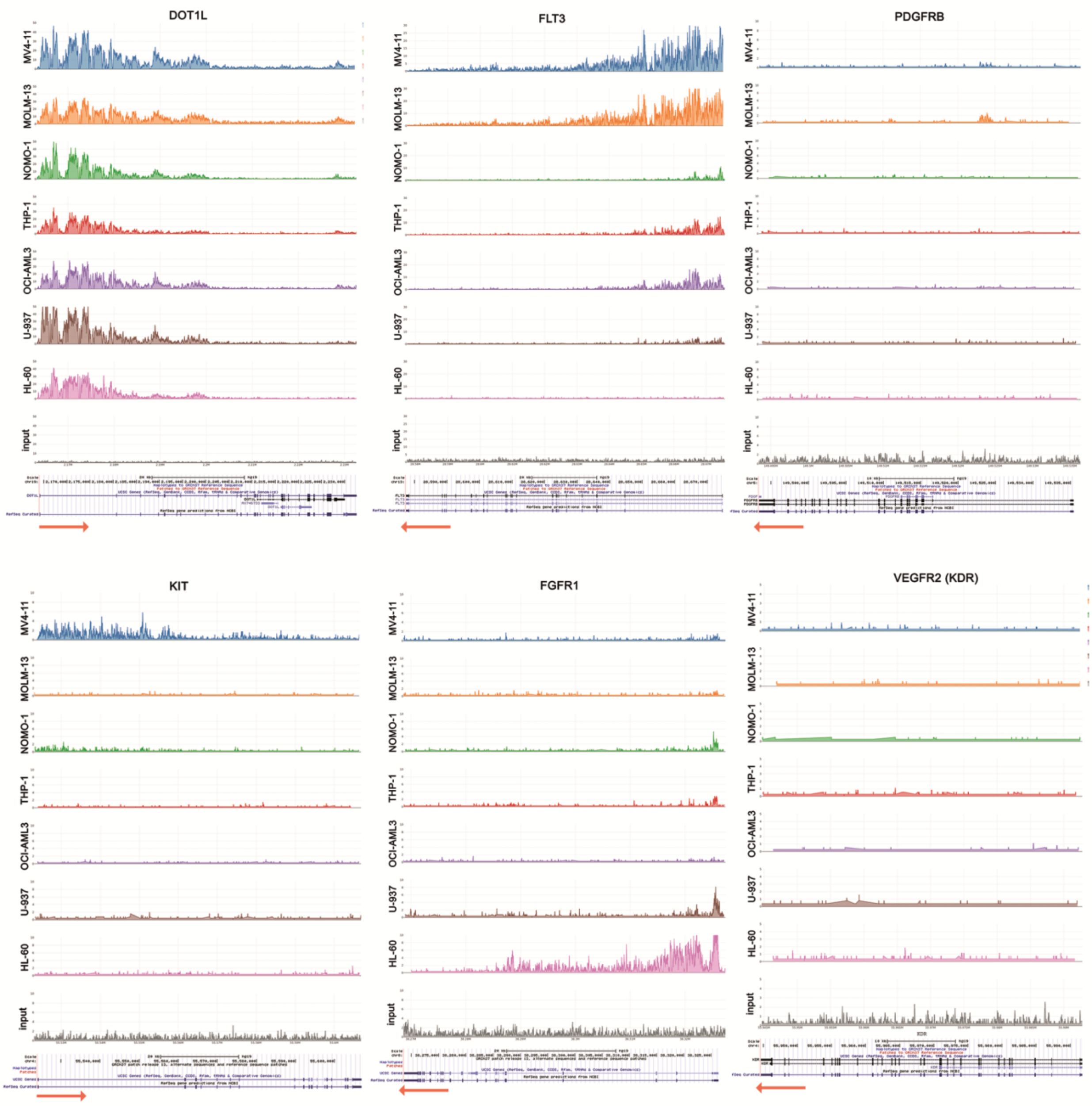

Figure S13: H3K79me2 profiles of DOT1L and Sorafenib target genes (*FLT3, PDGFRB*, *KIT*, *FGFR1* and *VEGFR2*) in *MLL*-r and non *MLL*-r human AML cell lines.

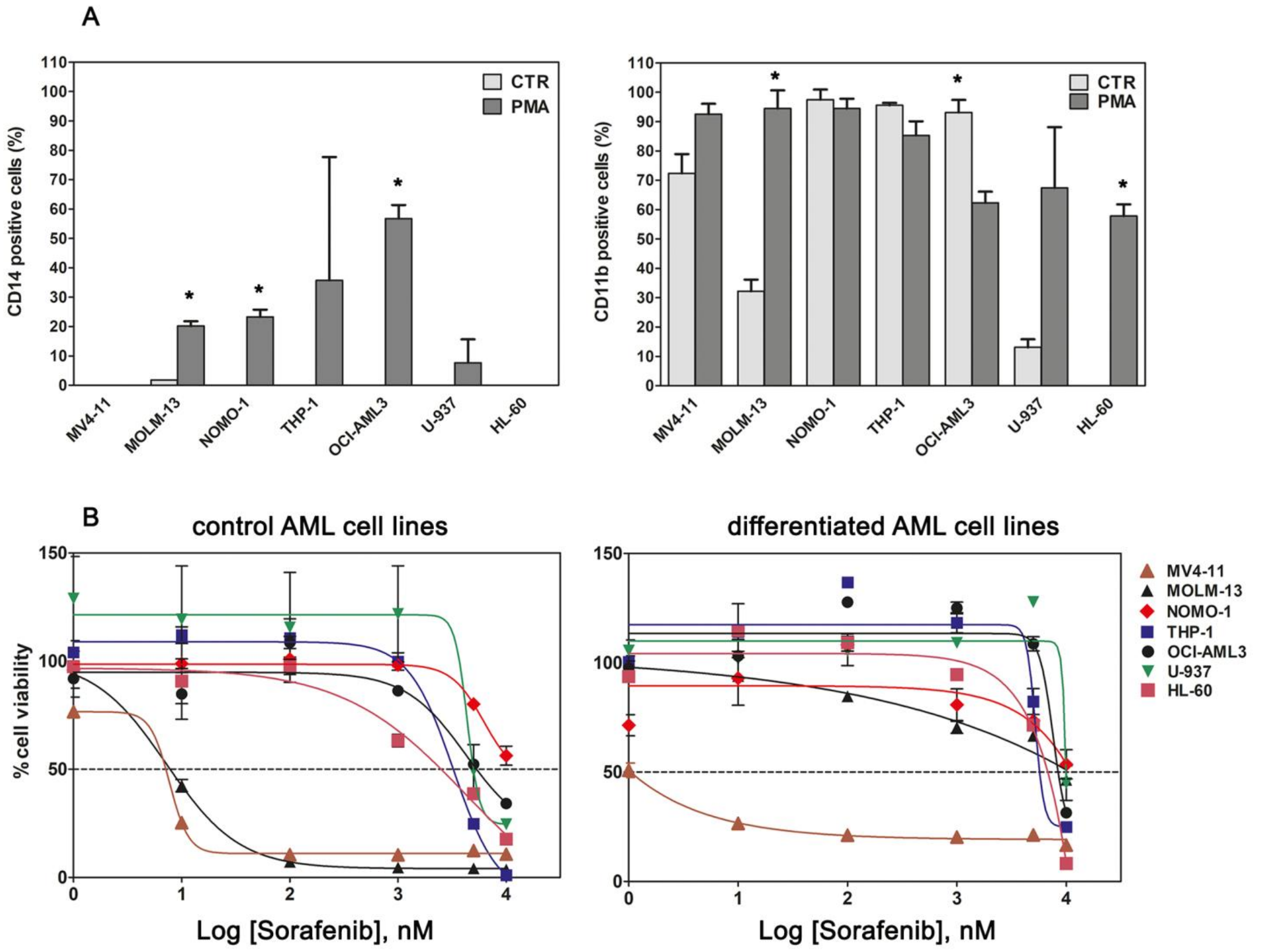

C

		control cells		differentiated cells		
	cell line	IC_{50} [μM]	SD	IC_{50} [μM]	SD	*p*
MLL-r	MV4-11	0.002	0.0004	<0.001	0.0004	0.02
	MOLM-13	0.024	0.34	9.75	0.34	0.04
	NOMO-1	>10	n.a.	>10	n.a.	ns
	THP-1	4.84	0.67	7.04	0.67	ns
non *MLL*-r	OCI-AML3	5.35	4.57	>10	4.57	ns
	U-937	5.99	4.48	>10	4.48	ns
	HL-60	3.81	3.24	7.44	3.24	ns

Figure S14: Assessment of the impact of differentiation stage in enhancing Sorafenib sensitivity. **a** AML cell lines were exposed to PMA to induce myelomonocitic differentiation, and flow cytometry analysis was performed to assess the differentiation antigens CD14 and CD11b expression, confirming AML cell maturation. Results of two independent replicates are presented as means ± SD. **b** MTT analysis of both undifferentiated (left panel) and differentiated (right panel) AML cells exposed to increasing Sorafenib concentration for 48 hours. **c** Comparison between IC_{50} values obtained in the control and differentiated group of AML cell lines. Results of two independent replicates are presented as means ± SD.

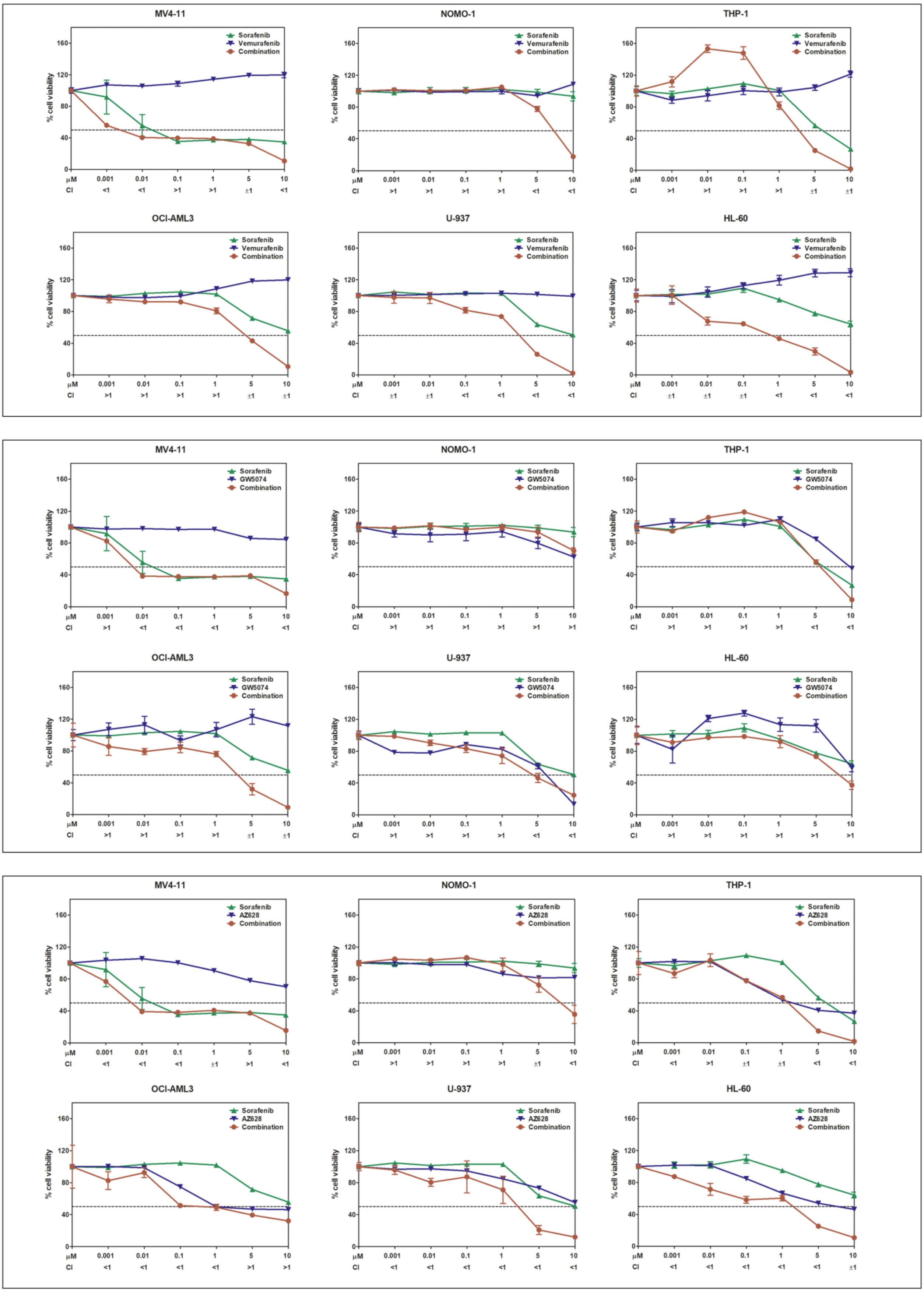

Figure S15: MTT analysis to assess the efficacy of Vemurafenib, GW5074 and AZ628 (BRAF, RAF1 and dual BRAF/RAF1 inhibitors, respectively) as single agents or in combination with Sorafenib. AML cell lines were treated with increased concentrations of the drugs for 24 hours. Drug combination ratio 1:1. Drug concentrations and combination indexes are shown at the bottom of each graph. Results of two independent replicates are presented as means ± SD.

Supplementary Tables

Table S1. AML cell lines characteristics. AML, acute myeloid leukemia; APL, acute promyleocytic leukemia

Cell line name	Derived from	Disease	Classification	Disease status	Genetic alterations
MV4-11	10-year-old boy	AML	FAB M5	Diagnosis	t(4;11) *MLL-AF4*; *FLT3*-ITD
MOLM-13	20-year-old man	AML	FAB M5a	Relapse	occult insertion ins(11;9)(q23;p22p23) resulting in *MLL-AF9; FLT3*-ITD; *CBL*^{mut}
NOMO-1	31-year-old woman	AML	FAB M5a	Relapse	t(9;11) *MLL-AF9*
THP-1	1-year-old boy	AML	-	Relapse	t(9;11) *MLL-AF9*
OCI-AML3	57-year-old man	AML	FAB M4	Diagnosis	*DNMT3A*^{R882C}
U-937	37-year-old man	histiocytic lymphoma	-	-	t(10;11) *CALM-AF10*
HL-60	35-year-old woman	APL	FAB M3	-	Complex karyotype
CMK	10-month-old boy	AMKL	FAB M7	Relapse	+21 (Down's syndrome)
WSU-AML	-	AML	FAB M7	-	-
KG1a	59-year-old man	AML	-	Relapse	expression of fusion gene FGFR1OP2-FGFR1
KASUMI-1	7-year-old boy	AML	FAB M2	Relapse	t(8;21) *RUNX1-RUNX1T1*; *KIT*^{N822}

Table S2. Clinical and genetic characteristics of patient analyzed in this study. BM, bone marrow; PB, peripheral blood.

Sample ID	Disease status	Material	Genetic alterations
#1	Diagnosis	BM	t(6;11) *MLL-AF6*
#2	Diagnosis	PB	t(10;11) *MLL-AF10*
#3	Diagnosis	BM	*FLT3*^{mut}
#4	Diagnosis	BM	*FLT3*-TKD; t(9;11) *MLL-AF9*
#5	Diagnosis	BM	t(10;11) *MLL-AF10*
#6	Refractory	-	t(10;11) *MLL-AF10*
#7	-	-	c-*KIT*^{mut}; t(8;21) *RUNX1-RUNX1T1*
#8	Diagnosis	PB	t(11;12)
#9	Diagnosis	BM	wt
#10	Diagnosis	PB	wt
#11	-	BM	*MLL* wt
#12	Diagnosis	-	*MLL* wt
#13	Diagnosis	-	*MLL* wt
#14	-	-	*MLL* wt
#15	Diagnosis	PB	*FLT3*^{mut}

Table S6. Primers used for ChIP qPCR analysis.

Primer Name	Distance from TSS	Forward Primer	Reverse Primer
Hoxa9_A	-1158 bp	TGCCTTTTCCCAAACCGAACT	TGAATCCCAGCTGGGGAAAAA
Hoxa9_B	TSS	CGTCGTTGGCCACAATTAAAA	TGTTTTTATGTAAAGGGATCG
Hoxa9_C	+0.5 kb	CTTGTGGTTCTCCTCCAGTTG	CTCATTCTCAGCATTGTTTTC
Meis1_A	-1.2 kb	CGACGATCATAAATAGCTTGG	AGGGAACAATGAGCTGAGCGC
Meis1_B	TSS	CCGGGGGAGTTTGAATATTTG	TCTCTGGCTCCCTTCCTACTT
Meis1_C	+2.3 kb	CGACGATCTACCCCATTACGG	TTCAGGTGGTGGACCGGCTGCAT

Supplementary References

1. Chou TC, Talalay P. Quantitative analysis of dose-effect relationships: the combined effects of multiple drugs or enzyme inhibitors. Adv Enzyme Regul. 1984;22:27-55. Epub 1984/01/01.
2. Lonetti A, Cappellini A, Sparta AM, Chiarini F, Buontempo F, Evangelisti C, et al. PI3K pan-inhibition impairs more efficiently proliferation and survival of T-cell acute lymphoblastic leukemia cell lines when compared to isoform-selective PI3K inhibitors. Oncotarget. 2015;6(12):10399-414. Epub 2015/04/15.
3. Lonetti A, Antunes IL, Chiarini F, Orsini E, Buontempo F, Ricci F, et al. Activity of the pan-class I phosphoinositide 3-kinase inhibitor NVP-BKM120 in T-cell acute lymphoblastic leukemia. Leukemia. 2014;28(6):1196-206. Epub 2013/12/07.
4. Lonetti A, Cappellini A, Bertaina A, Locatelli F, Pession A, Buontempo F, et al. Improving nelarabine efficacy in T cell acute lymphoblastic leukemia by targeting aberrant PI3K/AKT/mTOR signaling pathway. J Hematol Oncol. 2016;9(1):114. Epub 2016/10/26.
5. Schmittgen TD, Livak KJ. Analyzing real-time PCR data by the comparative C(T) method. Nat Protoc. 2008;3(6):1101-8. Epub 2008/06/13.
6. Ritchie ME, Phipson B, Wu D, Hu Y, Law CW, Shi W, et al. limma powers differential expression analyses for RNA-sequencing and microarray studies. Nucleic acids research. 2015;43(7):e47. Epub 2015/01/22.
7. Milne TA, Zhao K, Hess JL. Chromatin immunoprecipitation (ChIP) for analysis of histone modifications and chromatin-associated proteins. Methods Mol Biol. 2009;538:409-23. Epub 2009/03/12.
8. Lindgreen S. AdapterRemoval: easy cleaning of next-generation sequencing reads. BMC Res Notes. 2012;5:337. Epub 2012/07/04.
9. Langmead B, Salzberg SL. Fast gapped-read alignment with Bowtie 2. Nat Methods. 2012;9(4):357-9. Epub 2012/03/06.
10. Zhang Y, Liu T, Meyer CA, Eeckhoute J, Johnson DS, Bernstein BE, et al. Model-based analysis of ChIP-Seq (MACS). Genome Biol. 2008;9(9):R137. Epub 2008/09/19.
